# Coactosin-like protein 1 regulates integrity and repair of model intestinal epithelial barriers via actin binding dependent and independent mechanisms

**DOI:** 10.3389/fcell.2024.1405454

**Published:** 2024-07-08

**Authors:** Susana Lechuga, Armando Marino-Melendez, Austin Davis, Ajay Zalavadia, Afshin Khan, Michelle S. Longworth, Andrei I. Ivanov

**Affiliations:** Department of Inflammation and Immunity, Lerner Research Institute of Cleveland Clinic Foundation, Cleveland, OH, United States

**Keywords:** epithelial barrier, tight junctions, adherens junctions, actin cytoskeleton, wound healing, myosin II

## Abstract

The actin cytoskeleton regulates the integrity and repair of epithelial barriers by mediating the assembly of tight junctions (TJs), and adherens junctions (AJs), and driving epithelial wound healing. Actin filaments undergo a constant turnover guided by numerous actin-binding proteins, however, the roles of actin filament dynamics in regulating intestinal epithelial barrier integrity and repair remain poorly understood. Coactosin-like protein 1 (COTL1) is a member of the ADF/cofilin homology domain protein superfamily that binds and stabilizes actin filaments. COTL1 is essential for neuronal and cancer cell migration, however, its functions in epithelia remain unknown. The goal of this study is to investigate the roles of COTL1 in regulating the structure, permeability, and repair of the epithelial barrier in human intestinal epithelial cells (IEC). COTL1 was found to be enriched at apical junctions in polarized IEC monolayers *in vitro*. The knockdown of COTL1 in IEC significantly increased paracellular permeability, impaired the steady state TJ and AJ integrity, and attenuated junctional reassembly in a calcium-switch model. Consistently, downregulation of COTL1 expression in *Drosophila melanogaster* increased gut permeability. Loss of COTL1 attenuated collective IEC migration and decreased cell-matrix attachment. The observed junctional abnormalities in COTL1-depleted IEC were accompanied by the impaired assembly of the cortical actomyosin cytoskeleton. Overexpression of either wild-type COTL1 or its actin-binding deficient mutant tightened the paracellular barrier and activated junction-associated myosin II. Furthermore, the actin-uncoupled COTL1 mutant inhibited epithelial migration and matrix attachment. These findings highlight COTL1 as a novel regulator of the intestinal epithelial barrier integrity and repair.

## Introduction

The establishment of epithelial barriers is a hallmark of tissue morphogenesis resulting in the separation of internal organs from the environment and the formation of tissue compartments with unique chemical composition, cellular organization, and functions. Barrier assembly is driven by the adhesive interactions of epithelial cells mediated by adherens junctions (AJs) and tight junctions (TJs) (Meng and Takeichi, 2009; Troyanovsky, 2012; Van Itallie and Anderson, 2014; Mege and Ishiyama, 2017; Otani and Furuse, 2020; Balda and Matter, 2023). The strength of cell-cell adhesions and tightness of epithelial barriers are determined through adhesion bonds that are generated by transmembrane junctional proteins such as E-cadherin and claudins at AJs and TJs, respectively (Meng and Takeichi, 2009; Troyanovsky, 2012; Van Itallie and Anderson, 2014; Mege and Ishiyama, 2017; Otani and Furuse, 2020; Balda and Matter, 2023). However, adhesive interactions of these AJ and TJ molecules alone are insufficient to establish, sustain, and remodel intercellular contacts during epithelial morphogenesis and repair (Winklbauer, 2015; Lenne et al., 2021). Mechanical coupling of junctional proteins to the cortical actin cytoskeleton represents another key mechanism regulating the functions and dynamics of epithelial barriers (Maitre et al., 2012; Chan et al., 2017). Both AJs and TJs are connected to the cortical actin cytoskeleton by their scaffolding proteins which can interact with either actin or different actin-binding proteins. Alpha-catenin is the most well-known cytoskeletal linker at AJs (Troyanovsky, 2012; Mege and Ishiyama, 2017), whereas “zonula occludens” (ZO) proteins and cingulin mediate the cytoskeletal coupling of TJs (Citi, 2019; Varadarajan et al., 2019). AJs and TJs are associated with different forms of the cortical actin cytoskeleton. In well-differentiated epithelial cells, the most prominent perijunctional cytoskeletal structure is a circumferential belt composed of bipolar actin bundles running in parallel to the plasma membrane and attached to both AJs and the basal part of TJs (Hirokawa and Tilney, 1982; Yonemura et al., 1995; Ebrahim et al., 2013). Attachment of this circumferential belt to junctional complexes is likely to be mediated by orthogonal actin filaments extended towards the plasma membrane (Efimova and Svitkina, 2018; Lechuga and Ivanov, 2021; Li et al., 2021). The junction-associated actin cytoskeleton generates pushing and pulling mechanical forces applied to AJs and TJs that are essential for initiation, expansion, and disassembly of epithelial cell-cell contacts (Citi, 2019; Angulo-Urarte et al., 2020; Lenne et al., 2021; Campas et al., 2023). These forces are produced by the interplay between turnover of the perijunctional actin filaments and the filament contractility driven by a key actin motor, non-muscle myosin II (NM II) (Caorsi et al., 2016; Chan et al., 2017; Ito et al., 2017).

The structure and dynamics of actin filaments are controlled by a number of actin-nucleating, capping, bundling, and depolymerizing proteins (Pollard, 2016; Lappalainen et al., 2022; Rajan et al., 2023). Recent interactome studies have identified AJs and TJs as important molecular hubs enriched in various actin-binding and actin-regulating proteins (Van Itallie et al., 2013; Guo et al., 2014; Van Itallie et al., 2014; Fredriksson et al., 2015; Tan et al., 2020; Suarez-Artiles et al., 2022). While actin-dependent regulation of epithelial junctions attracted considerable attention, this field is primarily focused on the polymerization of actin filaments. For example, a large body of evidence supports the roles of the Arp2/3 complex (that nucleates branched actin filaments), in controlling junctional assembly in different epithelia (Verma et al., 2004; Ivanov et al., 2005; Kovacs et al., 2011; Verma et al., 2012; Han et al., 2014; Chanez-Paredes et al., 2021). In addition to the Arp2/3 complex, several formin proteins that nucleate linear actin filaments have been implicated in the regulation of AJ and TJ assembly (Carramusa et al., 2007; Ryu et al., 2009; Grikscheit et al., 2015; Rao and Zaidel-Bar, 2016). The involvement of other actin-binding proteins in the organization and functions of epithelial junctions remains poorly understood.

The actin-depolymerizing factor homology (ADF-H) domain proteins comprise an evolutionally conserved superfamily of critical actin regulators (Poukkula et al., 2011; Shishkin et al., 2016). There are five different subdivisions of this superfamily: ADF/cofilins, twinfilins, Abp1/drebrin, coactosins, and glial maturation factors (Poukkula et al., 2011; Shishkin et al., 2016). While the primary sequence of their ADF-H domains only shares 20% similarity, the basic fold of this domain is remarkably similar in all proteins of this superfamily, consisting of five internal beta-strands and four peripheral alpha-helices (Poukkula et al., 2011; Hild et al., 2014). This structure enables interactions with monomeric (G) and filamentous (F)-actin, as well as the Arp2/3 complex (Poukkula et al., 2011; Hild et al., 2014). Different members of the ADF-H domain superfamily are best known for disrupting actin filaments by either promoting filament disassembly, attenuating filament assembly, or Arp2/3 inhibition (Poukkula et al., 2011; Shishkin et al., 2016; Lappalainen et al., 2022). Due to their roles in regulating F-actin turnover, ADF-H domain proteins have been implicated in controlling important actin-dependent cellular processes, such as cell migration, vesicle trafficking, cell polarity, and cytokinesis (Poukkula et al., 2011; Shishkin et al., 2016). Little is known about the roles of ADF-H domain proteins in regulating epithelial junctions, with published studies in this field primarily focusing on members of the ADF/cofilin subfamily. Thus, both ADF and cofilin-1 were shown to control TJ integrity and permeability in the intestinal epithelium and vascular endothelium *in vitro* and *in vivo* (Wang et al., 2012; Shi et al., 2016; Wang et al., 2016; Maldonado-Contreras et al., 2017). Yet, the importance of other ADF-H domain proteins for the assembly and function of epithelial junctions remains poorly investigated.

Coactosin-like protein 1 (COTL1) is the least studied member of the ADF-H domain protein superfamily, despite having unique cellular activities. This small (∼ 17 kDa) protein selectively binds to actin filaments, but not to monomeric actin (de Hostos et al., 1993; Provost et al., 2001a). However, unlike other ADF-H domain proteins, COTL1 and its low eucaryotic paralogs do not trigger actin filament disassembly (de Hostos et al., 1993; Provost et al., 2001a), instead in some systems they could stabilize actin filaments and protect them from depolymerization (Kim et al., 2014; Kumar et al., 2014). In addition to actin filament binding, COTL1 interacts with 5-lipooxygenase (5-LO) to regulate leukotriene biosynthesis (Provost et al., 2001b; Rakonjac et al., 2006). Moreover, recent studies suggest another noncanonical activity of COTL1 that involves inhibition of the TGF-β signaling pathway (Xia et al., 2018; Zhang et al., 2021). Given the multiple molecular interactions and activities of COTL1 as described above, one can suggest it has many important cellular functions. However, functional roles of this protein, remain poorly defined. Several previous studies implicated COTL1 in the regulation of morphogenic cell movement during normal development (Hou et al., 2013; Li et al., 2018; Liu et al., 2018; Hou et al., 2021) and controlling metastatic dissemination of cancer cells (Guo et al., 2017). These studies were performed in different experimental systems and yield conflicting data that suggests either promigratory (Hou et al., 2013; Guo et al., 2017) or antimigratory roles (Li et al., 2018; Liu et al., 2018) of COTL1. Furthermore, molecular mechanisms underlying the described effects of COTL1 on cell motility remain unknown. While COTL1 was found to regulate immune cell interactions and formation of the immunological synapse (Kim et al., 2014; Bolger-Munro et al., 2021), no previous studies addressed the importance of this actin-binding protein for the structure and permeability of epithelial junctions. The present study is designed to close this knowledge gap and examine whether COTL1 plays a role in regulating the integrity and repair of model intestinal epithelial barriers. Our results suggest that COTL1 is essential for the assembly of AJs and TJs and the establishment of the epithelial barrier, and it also controls collective epithelial cell migration and attachment to the extracellular matrix (ECM). Most interestingly though, not all observed functions of COTL1 require its interactions with actin filaments.

## Materials and methods

### Antibodies and other reagents

The following monoclonal (mAb) and polyclonal (pAb) antibodies were used to detect apical junction, and cytoskeletal proteins: anti-E-cadherin (BD Biosciences, San Jose, CA, Cat# 610181), anti-β-catenin (BD Biosciences, Cat# 610154), anti-claudin 4 (Thermo Fisher Scientific, Waltham, MA, Cat# 32–9,400) and anti-turbo GFP (Origene Technologies Rockville, MD, Cat# TA150041) mAbs; anti-NM IIA (Biolegend, San Diego, CA, Cat# 909801), anti-phosphorylated Myosin Light Chain 2 (Ser19) (Cell Signaling, Danvers, MA, Cat# 3671), anti-ZO-1, anti-claudin 1 (Thermo Fisher Scientific, Cat# 40–2,200 and 51–9,000), anti-occludin (Proteintech, Rosemont, IL, Cat# 13409-1-AP), anti-COTL1 (Proteintech, Cat# 10781-1-AP), and anti-E-cadherin (R and D System, Minneapolis, MN, Cat# AF748) pAbs. Coralite Plus 488-conjugated anti-COTL1 monoclonal antibody (Proteintech, Cat# CL488-60237) and Alexa Fluor-555-conjugated Phalloidin (Thermo Fisher Scientific, Cat# A43055). Alexa Fluor-488-conjugated donkey anti-rabbit (Cat# A-21206), Alexa Fluor-555-conjugated donkey anti-mouse (Cat# A-31570), and Alexa Fluor-488-conjugated donkey anti-goat (Cat# A-11055) secondary antibodies were obtained from Thermo Fisher Scientific. Horseradish peroxidase-conjugated goat anti-rabbit (Cat# 170–6,515) and anti-mouse (Cat# 170–6,516) secondary antibodies were obtained from Bio-Rad Laboratories (Hercules, CA). Latrunculin B and other reagents were obtained from Millipore-Sigma (Burlington, MA).

#### *Drosophila* stocks

The UAS-mCherry dsRNA stock (#35785) was obtained from the Bloomington *Drosophila* Stock Center. The UAS-cotl1 dsRNA stock (v109738) was obtained from the Vienna *Drosophila* Resource Center. The esgGAL4 stock was a generous gift from Dr. Norbert Perrimon (Harvard University, Boston, MA). All stocks were maintained at 25°C on the standard dextrose medium, prior to experimentation.

#### Cell culture

SK-CO15 human colonic epithelial cells were a gift from Dr. Enrique Rodriguez-Boulan (Weill Medical College of Cornell University, New York, NY). DLD1 (Cat# CCL-221) and Caco-2 (Cat# HTB-37) human colonic epithelial cell lines were obtained from the American Type Culture Collection (ATCC, Manassas, VA). All cell lines were authenticated by the small tandem repeat profiling by LabCorp (Burlington, NC), were mycoplasma-free according to the *mycoplasma* PCR detection assay (PromoCell, Heidelberg, Germany, Cat# PK-CA20-700–20) and used until passage 10. DLD1, SK-CO15 and Caco-2 cells were cultured in DMEM with high glucose and low bicarbonate (ATCC, Cat# 30–2002) medium supplemented with 10% fetal bovine serum (FBS), HEPES, non-essential amino acids, and penicillin-streptomycin antibiotic. The cells were seeded on collagen-coated Transwell membrane filters or glass coverslips for permeability measurements and immunolabeling experiments respectively. For the biochemical studies, the cells were plated into six well cell culture plates.

#### Calcium switch assay

The calcium switch assay was performed as previously described (Gonzalez-Mariscal et al., 1990; Ivanov et al., 2005; Ivanov et al., 2007; Lechuga et al., 2015; Lechuga et al., 2022). Briefly, well-differentiated DLD1 and SK-CO15 cell monolayers were subjected to overnight extracellular calcium depletion by incubating in the Eagle’s minimum essential medium for suspension culture (ATCC, Cat# 11380) supplemented with 5 µM CaCl_2_, 10 mM HEPES and 10% dialyzed FBS, pH 7.4. To induce junctional reassembly, the cells were returned to a normal cell culture medium with high (1.8 mM) calcium for the indicated times.

#### Small-interference RNA-mediated knockdown of COTL1 in IEC

The small interference (si) RNA-mediated transient knockdown of COTL1 in DLD1 and SK-CO15 IEC was carried out by using FlexiTube siRNA duplexes obtained from Qiagen (Hilden, Germany), as previously described (Babbin et al., 2009; Lechuga et al., 2015). The following sequences were selected to target human COTL1: COTL1 siRNA 1 (Hs_COTL1_4, Cat# SI00352058), 5′-AAG​GAG​TTT​GTG​ATC​AGT​GAT-3’; COTL1 siRNA 2 (Hs_COTL1_5, Cat# SI04140220); 5′-CCG​GAG​TTG​CCT​AAG​ATG​CAT-3’; COTL1 siRNA 3 (Hs_COTL1_6, Cat# SI04141732), 5′-CTG​TAC​TGT​ATT​GGG​ATT​GTA-3’; and COTL1 siRNA 4 (Hs_COTL1_7, Cat# SI04363653, 5′-CTC​CGC​GGC​GAT​GGC​CAC​CAA-3’. A scrambled siRNA sequence, lacking complementarity to any human gene, was used as control siRNA: 5′-AAT​TCT​CCG​AAC​GTG​TCA​CGT-3’ (Ctr_Control_1, Cat# SI03650352).

IEC were transfected using DharmaFECT 1 reagent (Horizon, Waterbeach, United kingdom, Cat# T-2001–02) in Opti-MEM I medium (Thermo Fisher Scientific, Cat# 31985070), according to the manufacturer’s protocol, with a final siRNA concentration of 50 or 100 nM. Cells were utilized for experiments on day 4 post-transfection.

#### Plasmid construction and site-directed mutagenesis

Stable DLD1 cell lines were generated to express COTL1 wild type tagged with turbo GFP (tGFP-COTL1 WT), and its mutants unable to bind either F-actin, (tGFP-COTL1 R73E/K75E), or 5-LO (tGFP-COTL1 K131A). The construction of pCMV6-AC-tGFP, a mammalian vector with C-terminal turbo GFP, containing COTL1 WT was obtained from Origene Technologies (Cat# RG204055). The QuikChange Multi Site-Directed Mutagenesis Kit (Agilent Technologies, Santa Clara, CA, Cat# 200515) was utilized to create tGFP-COTL1 R73E/K75E and tGFP-COTL1 K131A mutants. The following phosphorylated primers were used: R73E/K75E: 5′-GGG​ATG​CCA​TGA​GCA​AGG​AGT​CCG​AGT​TTG​CCC​TCA​TCA​CG-3′ and K131A 5′- CGT​GAT​GAG​GGC​AAA​CTC​GGA​CTC​CTT​GCT​CAT​GGC​ATC​CC-3′ in the PCR mutagenesis reaction performed according to the manufacturer’s protocol using a PCR C1000 Touch Thermal Cycler (Bio-Rad). The amplificated products were digested with the restriction endonuclease DpnI to eliminate the methylated and hemimethylated parental DNA template, and then the reaction mixture was transformed into XL10-Gold^®^ ultracompetent cells. Double-stranded plasmid DNA was then purified from the transformants with the Pure-Yield Miniprep System (Promega, Madison, WI, Cat# A1222). The presence of mutation was corroborated by DNA sequencing analyses.

The wild-type and mutant tGFP-COTL1 constructs were transfected into DLD1 cells using TransIT®-LT1 Transfection Reagent (Mirus Bio, Madison, WI, Cat# MIR 2304) as indicated by the manufacturer. 48 h after transfection, fresh medium containing G418 antibiotic (0.5 mg/mL) was added. After 7 days of antibiotic selection, tGFP-expressing cells were sorted using the FACSAria™ fusion flow cytometer (BD Bioscience). Briefly, cultured cells were harvested and resuspended in PBS with 1% of FBS for the sorting. After sorting, cells were collected in DMEM with 10% FBS and the overexpression of tGFP-COTL1 WT and its mutants was analyzed by immunoblotting. The sorted DLD1 cells overexpressing tGFP-COTL1 constructs or control tGFP were used as mixed populations without single clone isolation.

#### Measurement of epithelial barrier permeability

For the transepithelial electrical resistance (TEER) and FITC-dextran flux measurements, 6 × 10^4^ of DLD1 or SK-CO15 cells were plated on collagen I-coated Transwells (Falcon, Corning, NY, Cat# 353095) with polyethylene terephthalate membrane filters (0.4 µm pore size). TEER was measured using an EVOM2 Volt Ω Meter (World Precision Instruments, Sarasota, FL). The resistance of cell-free collagen-coated filters was subtracted from each experimental point. A transmonolayer FITC-dextran flux assay was performed as previously described (Lechuga et al., 2015; Lechuga et al., 2022). IEC monolayers cultured on Transwell filters were apically exposed to 1 mg/mL of FITC-labeled dextran (4,000 Da) in HEPES-buffered Hanks’ Balanced Salt Solution with calcium and magnesium (HBSS^+^). After 120 min of incubation, samples were collected from the lower chamber, and FITC fluorescence intensity was measured using a BioTek Synergy H1 microplate reader (Agilent Technologies), at excitation and emission wavelengths 485 nm and 544 nm, respectively. The amount of FITC-dextran translocated across the epithelial cell monolayer was calculated based on a calibration curve using Prism 10 software (GraphPad, La Jolla, CA).

#### Immunoblotting analysis

IEC monolayers were scraped and homogenized using a Dounce homogenizer in RIPA buffer (20 mM Tris, 50 mM NaCl, 2 mM EDTA, 2 mM EGTA, 1% sodium deoxycholate, 1% Triton X-100 (TX-100), and 0.1% SDS, pH 7.4) containing protease inhibitor cocktail, phosphatase inhibitor cocktails 2 and 3, and Pefabloc^®^ (Millipore-Sigma, Cat# P8340, P5726, P0044, and 76,307 respectively). The obtained total cell lysates were cleared by centrifugation (20 min at 14,000 x g), diluted with 2x Laemmli sample loading buffer, and boiled. SDS-polyacrylamide gel electrophoresis was conducted using a standard protocol with equal amounts (10 μg or 20 μg) of total protein loaded per lane. The separated proteins were transferred to nitrocellulose membranes and the membranes were then blocked with 5% non-fat milk. The blocked membranes were incubated overnight with primary antibodies at 1:500 or 1:1,000 dilution, and then exposed for 1 h to HRP-conjugated secondary antibodies at 1:5,000 dilution. The immunolabelled proteins were visualized using a standard enhanced chemiluminescence solution and X-ray films. Band intensities were quantified using the gel densitometry toolset from ImageJ 1.51K software (National Institute of Health, Bethesda, MD).

#### Immunofluorescence labeling, confocal microscopy, and image analysis

To visualize the structure of epithelial junctions, the localization of COTL1, and NM II, cultured IEC monolayers were fixed and permeabilized with 100% ice-cold ethanol for 20 min at −20°C. To visualize phosphorylated myosin light chain (pMLC), cells were fixed with 4% paraformaldehyde (PFA) for 20 min and permeabilized with 0.5% Triton X-100 in HBSS^+^ for 5 min at room temperature. Fixed samples were blocked for 60 min in a blocking buffer (HBSS^+^ containing 1% bovine serum albumin, pH 7.4) followed by a 60 min incubation with primary antibodies diluted in the blocking buffer (1:200 dilution). Then, samples were washed three times with the blocking buffer, incubated with Alexa-Fluor-488–conjugated donkey anti-rabbit and Alexa-Fluor-555–conjugated donkey anti-mouse secondary antibodies at 1:1,000 dilution in the blocking buffer for 60 min, rinsed three times with the blocking buffer, and mounted on slides with ProLong™ Gold Antifade mounting medium with DNA stain DAPI (Thermo Fisher Scientific, Cat #P36941). For the actin cytoskeleton labeling, fixed/permeabilized cells were incubated for 1 h with Alexa-Fluor-488 or Alexa-Fluor-555 conjugated phalloidin.

Fluorescence labeled IEC monolayers were imaged using Leica 100x CS2 (1.4NA) OIL immersion objective and Leica TCS SP8 AOBS confocal laser scanning system attached to a Leica DMi8 inverted epifluorescence microscope (Wentzler, Germany). The Alexa Fluor 488 and 555 signals were acquired sequentially in frame-interlace mode, to eliminate cross-talk between channels. The Alexa 488 fluorophore and tGFP were excited with the 488 nm laser line of an Argon laser at 15%–30% power, and the Alexa 555 fluorophore was excited with the 561 nm laser line of a DPSS 561 laser at 5%–15% power. The *xy* and *xz* optical images were collected with the pixel size of 0.11 µm and 0.05 µm, respectively. For acquiring *xy* images of the steady-state differentiated IEC monolayers with wavy apical surface, the representative regions of control cell monolayers and the areas with markedly decreased COTL1 labeling in COTL1-depleted cell monolayers were selected. At the selected areas, 4–6 sections were taken to make a small *z*-stack covering a physical length of 2–3 µm. The start and the end positions of the sections were selected to include the apical junctional complex and the perijunctional actomyosin belt. For imaging of the calcium repletion experiments with flattened cells, single sections at the level of most intensive signal at the cell-cell contact areas were acquired.

To quantify ZO-1 accumulation at the multicellular junctions, the images were imported into ImageJ 1.51K, and background was similarly subtracted for each image. The signal intensities of small circle area manually drawn around the tricellular/multicellular junction and the same area placed at the adjacent bicellular junction were measured, and the ratio of the multicellular/bicellular signal intensities were calculated. Fifty different ratios calculated for each image were averaged to yield a single data point. Four different images were quantified for each experimental group. To quantify the pMLC signal intensity, the images were imported into ImageJ 1.51K, and the signal intensity of a rectangular area manually drawn over the pMLC labeled junctional region was measured. Twelve different intensity measurements per image were averaged to yield a single data point. Three different images were quantified for each experimental group. For quantifying the junctional length in the calcium repletion experiments, original images were imported into ImageJ 1/51K, adjusted with a binary thresholding and their background was subtracted. The total length of junctional labeling was measured manually, and the obtained values were normalized by the number of cell nuclei per microscopic field. Four different images were quantified for each experimental group. Junctional length measurement was performed in a blinded fashion by an independent investigator not involved in image acquisition. To quantify cell height, xz sections were imported into ImageJ 1.51K, a line from the most apical point (ZO-1 signal) to the basal region was manually drawn. The height of 8 cells per image were measured and averaged to yield a single data point. Four different images were quantified for each experimental group.

For cell size measurement, the images were prepared by making a maximum intensity projection from a small z-stack captured through the monolayer of cells. Initially the cells were detected using a deep learning-based method CellPose (v3.0.8). We used the pretrained model “cyto3” with a diameter of 100, flow threshold 0.6 and cell probability threshold of −1 for the detection of cells. The results of the initial segmentation were manually corrected for any errors, to add missing cells, and, to remove incomplete cells on the edge of the images. ROIs of the cells were imported and measured in FIJI distribution of ImageJ (v1.53t). Total cell numbers were 385, 189, and 204 for controls siRNA, COTL1 siRNA1 and COTL1 siRNA3 groups, respectively. Four different images were quantified for each experimental group.

#### Wound closure assay

IEC were plated on either 6-well plates or Culture-Insert 3 Well in µ-Dish 35 mm (Ibidi GmbH, Gräfelfing, Germany, Cat# 80366) and allowed to grow to confluence. Wounding was done by either scratching cell monolayers with a pipette tip on 6-well plates or removing a 3-well silicone gasket in the dish to create well-defined 500 μm cell-free gaps. The bottom of the well or dish was marked to define several exact positions along the wound’s edge. The monolayers were supplied with fresh cell culture medium and images of a cell-free area at the marked regions were acquired at 0 h and at the indicated times after wounding using a Keyence BZ-X700 microscope (Keyence, Osaka, Japan) with the Keyence PlanApo λ 10 × 0.45/4. mm objective. The wound area was measured at three different positions using ROI manager tools from ImageJ 1.51K and the obtained numbers were averaged to create a single data point. Four different wounds were quantified for each experimental group.

#### Live cell imaging and data analysis

For live cell imaging, tGFP-COTL1 WT and tGFP-COTL1 R73E/K75E cells were grown in a coverslip bottom dish with 4 compartments, µ-Dish 35 mm Quad (Ibidi GmbH, Gräfelfing, Germany, Cat# 80416). Once the monolayer was achieved a scratch was made using a pipette tip in each of the quadrants. Multiple locations were identified along the edge to locate the cells with characteristics of migration. A Leica 63x CS2 (1.4NA) OIL immersion objective and Leica TCS SP8 AOBS confocal laser scanning system attached to a Leica DMi8 inverted epifluorescence microscope and equipped with a stage top incubator (OKOLAB United States Inc. Sewickley, United States) were used for live cell imaging. Argon laser was set to 30% power during the startup and the final laser power for the 488 nm laser line was kept low using AOBS (1.5%) during scanning. A scanning speed of 600 Hz allowed us to achieve a low pixel dwell time of 0.4 µs We did not directly measure the effect of phototoxicity on cells; however, a DIC scan along with the fluorescence was also captured allowing us to see overall cell morphology. The combination of low laser power, low pixel dwell time, and 1-minute intervals allowed us to keep the effect of phototoxicity to a minimum while permitting the capture of very dynamic movements of COTL1. The FIJI distribution of ImageJ (v 1.53t) was used to apply the median filter to timelapse images and for making the final movies. Time-lapse images for multiple positions across the edge of the scratch were captured at 1-minute intervals for 6 h. This experiment was repeated 3 times, and the representative field of views was selected for generating the movie. A median filter was applied to the time-lapse images to reduce the noise before exporting them as AVI.

#### Boyden chamber migration assay

Boyden chamber migration assay was performed using Transwell 6.5 mm membrane inserts with 8.0 µm pores (Falcon, Cat# 353097) that were coated with 15 μg/cm^2^ of collagen I. Cells were detached from the plate using a TrypLE Express solution (Thermo Fisher Scientific, Cat#12605), resuspended in serum-free medium, and added to the Transwell upper chamber at the density of 5,000 SK-CO15 cells per chamber. A complete cell culture medium containing 10% FBS as a chemoattractant was added to the lower chamber and cells were allowed to migrate for 16 h at 37°C. Membrane inserts were fixed with 100% methanol at room temperature for 10 min and non-migrated cells were removed from the top of the filter using a cotton swab. The cells that remained at the bottom of the filter were labeled with ProLong™ Gold Antifade mounting medium with DAPI and resulting images were taken using the Keyence BZ-X700 microscope with the Keyence PlanApo λ 10 × 0.45/4.mm objective and a BZ-X filter DAPI. Three images per membrane insert were taken and the number of DAPI-positive cells in each image was counted using the ImageJ 1.51K and averaged to yield a single data point.

#### Extracellular matrix attachment assay

Cells were detached from the plate using a TrypLE Express solution, counted with a T20™ automated cell counter (Bio-Rad), and resuspended in the complete medium. DLD1 and SK-CO15 cell suspensions containing 15,000 and 10,000 cells respectively, were seeded into a rat collagen I coated 24-well plate and were allowed to adhere for 30 and 20 min respectively, at 37 C. After incubation, unattached cells were aspirated, and the wells were gently washed with HBSS^+^ buffer. The attached cells were fixed with 100% methanol at room temperature for 10 min and stained using a Differential Quick III Staining Kit (Electron Microscopy Sciences, Hatfield, PA, Cat# 26096). Images of adherent cells were captured using the Keyence BZ-X700 microscope with the Keyence PlanApo λ 10 × 0.45/4.mm objective. Three images were taken for each well and the number of adhered cells in each image was counted using the ImageJ 1.51K and averaged to yield a single data point.

#### *Drosophila* smurf assay

Flies were aged on standard medium for 50 days post-eclosion, before performing the Smurf assay. 2.5% (wt/vol) Brilliant Blue FCF dye solution (Millipore-Sigma, Cat# 80717) was prepared in 5% sucrose solution. Small pieces of Kimwipes were saturated with 200 µL dye solution and placed into empty vials. 25 female flies were placed into each vial for 24 h, and each vial of 25 female flies was considered a biological replicate. At least three biological replicates were analyzed per genotype. A Smurf fly was counted when total body dye coloration was observed after 24 h. A fly exhibiting blue dye in part of the abdomen but not the entire body was not considered to be Smurf positive. A Leica S6 E Greenough stereomicroscope with 6.3:1 zoom was used to sort and identify Smurf flies. The average percentage of Smurf flies per vial was counted for a total of five vials per genotype. Statistical significance was calculated using the Mann-Whitney test.

#### Quantitative RT-PCR analysis

25 adult female intestines were dissected per biological replicate and submerged in cold PBS on ice. PBS was removed and replaced with Trizol. Tissues were dissected, submerged in Trizol (Life Technologies) in a microcentrifuge tube, and homogenized using a Pellet pestle cordless motor (Kimble) and disposable Kontes pestle. RNA was extracted during the standard Trizol protocol, according to the manufacturer. Extracted RNAs were then treated with RNase-free DNase in buffer RDD (Qiagen) prior to further purification using a RNeasy Mini Kit (Qiagen). An aliquot of cDNA was generated from 0.5–2 µg of total RNA using the TaqMan Reverse Transcription Reagents (Applied Biosystems) and an oligo-dT (16) primer (Invitrogen). Quantitative RT-PCR was performed using the Roche Lightcycler 480 to amplify 15 µL reactions containing 0.5 µL of cDNA, 0.5 µL of a 10 µM primer mix and 7.5 µL of 2X Fast Start SYBR Green Master Mix (Roche). Each reaction was performed in triplicate. Crossing point (Cp/Ct) values were determined using the Roche LightCycler 480 Absolute Quantification Second Derivative Analysis software. Relative quantitation of transcript levels was then performed using the delta Ct method (2^−ΔΔCT^) where the Ct values of a reference gene (rp49) in each sample are subtracted from the Ct values of the gene of interest to create a ΔCt value for each sample. The ΔCt is compared to a control sample to generate a ΔΔCt value for each sample. Following calculation of 2^−ΔΔCT^ for each sample, triplicates were averaged. Three, independent biological replicates were performed in all experiments, and the results of the three assays were averaged together, and standard deviations were calculated. The sequences of oligos used in the qRT-PCR studies are listed below:

COTL Fwd 5′-TAG​AGA​TGC​CAG​AGC​CCA​GA-3′ and COTL Rev 5’-ACG​GAA​GAG​CTC​AAT​GTC​CA-3’; rp49 Fwd 5′-AGG​GTA​TCG​ACA​ACA​GAG​TG-3′ and rp49 Rev 5′-CAC​CAG​GAA​CTT​CTT​GAA​TC-3’.

#### Statistical analysis

The data are expressed as means ± standard error (SEM). The statistical analysis was performed by using a two-tailed unpaired Student’s -test to compare the results obtained with two experimental groups. When the assumption of Gaussian distribution was not met, a non-parametric Mann-Whitney *U*-test was used for comparison. When more than two samples were compared, a one-way ANOVA with Bonferroni’s *post hoc* test was used (to compare overexpression tGFP-COTL1 WT and tGFP-COTL1 R73E/K75E; with the tGFP control). *p* values < 0.05 were considered statistically significant and all statistical analysis was performed using GraphPad Prism 10.

## Results

### COTL1 depletion increases paracellular permeability, disrupts steady-state junctional integrity, and attenuates TJ and AJ reassembly

**FIGURE 1:**
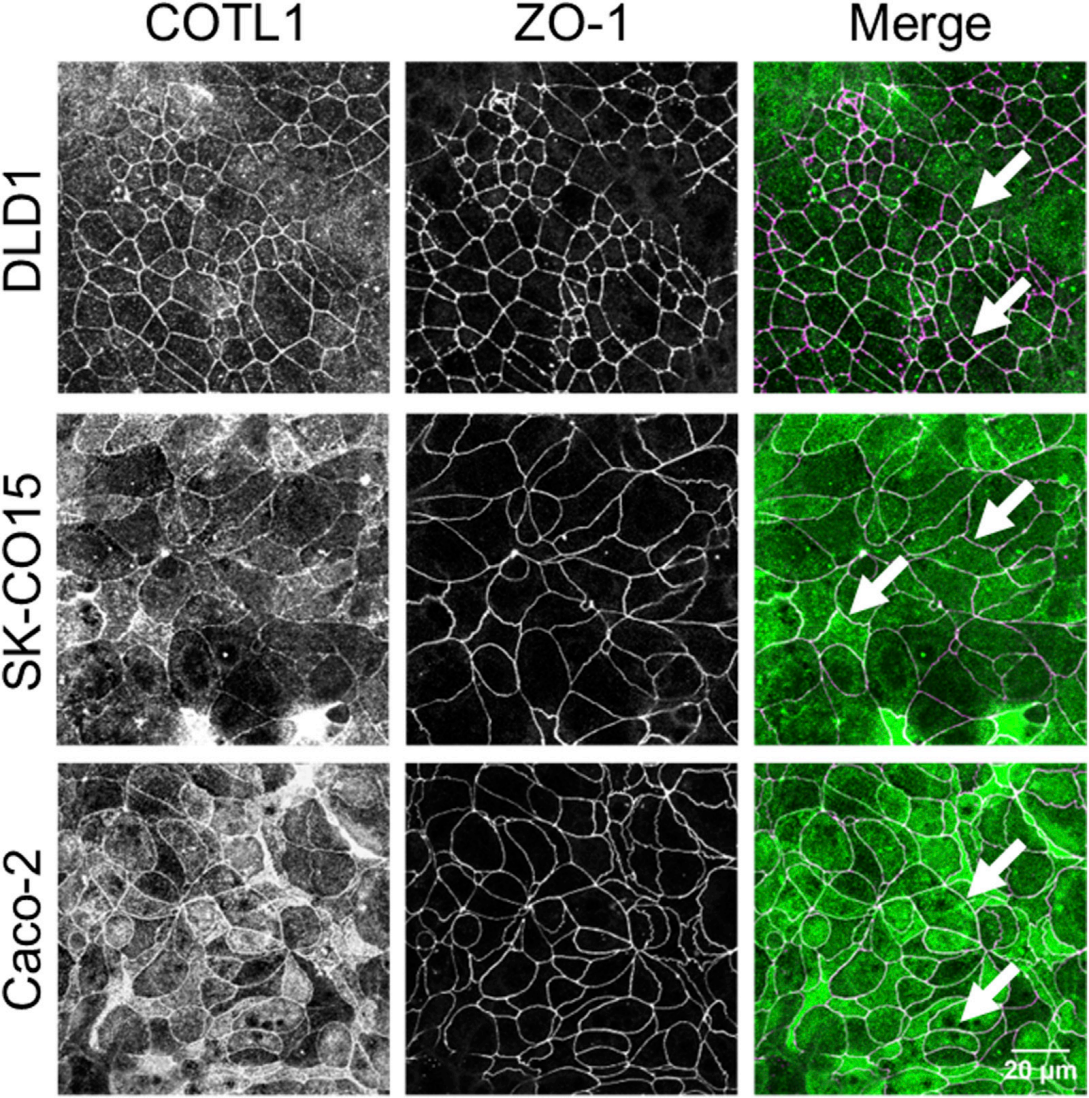
COTL1 localizes at tight junctions in polarized intestinal epithelial cells. Dual-immunofluorescence labeling of COTL1 (green) and ZO-1 (magenta) in confluent, polarized DLD1, SK-CO15, and Caco-2 epithelial cell monolayers. Arrows point at COTL1 localization at TJs. Images shown are representative of three independent experiments with multiple images taken per slide.

Since various actin regulators essential for the establishment of epithelial barriers are commonly enriched at AJs and/or TJs (Verma et al., 2004; Ivanov et al., 2005; Ryu et al., 2009; Han et al., 2014; Grikscheit et al., 2015; Lechuga et al., 2015; Rao and Zaidel-Bar, 2016) we first investigated if COTL1 is also localized at epithelial apical junctions. A dual-immunofluorescence labeling and confocal microscopy showed colocalization of COTL1 with a TJ marker, ZO-1 in three different human colonic epithelial cell lines (Figure 1, arrows), which was especially prominent in DLD1 and Caco-2 cells. Analysis of the *xz* projections of the confocal images showed a focal enrichment of COTL1 at the apical aspect of the plasma membrane, where it colocalized with both TJ and AJ markers (Supplementary Figure S1, arrows). Next, we examined COTL1’s association with the junctional actin cytoskeleton. In differentiated DLD1 cell monolayers, COTL1 was colocalized with the perijunctional F-actin belt (Supplementary Figure S2A, arrow). Actin filament depolymerization by Latrunculin B resulted in the disruption of the perijunctional COTL1 labeling (Supplementary Figure S2A, arrowhead). Furthermore, junctional labeling of COTL1 disappeared in IEC subjected to overnight extracellular calcium depletion but was restored at 4–6 h after calcium repletion following reassembly of the perijunctional F-actin belt (Supplementary Figure S2B, arrows). Together these data suggest the specific enrichment of COTL1 at the apical junctional complex and its association with the circumferential F-actin belt.

**FIGURE 2:**
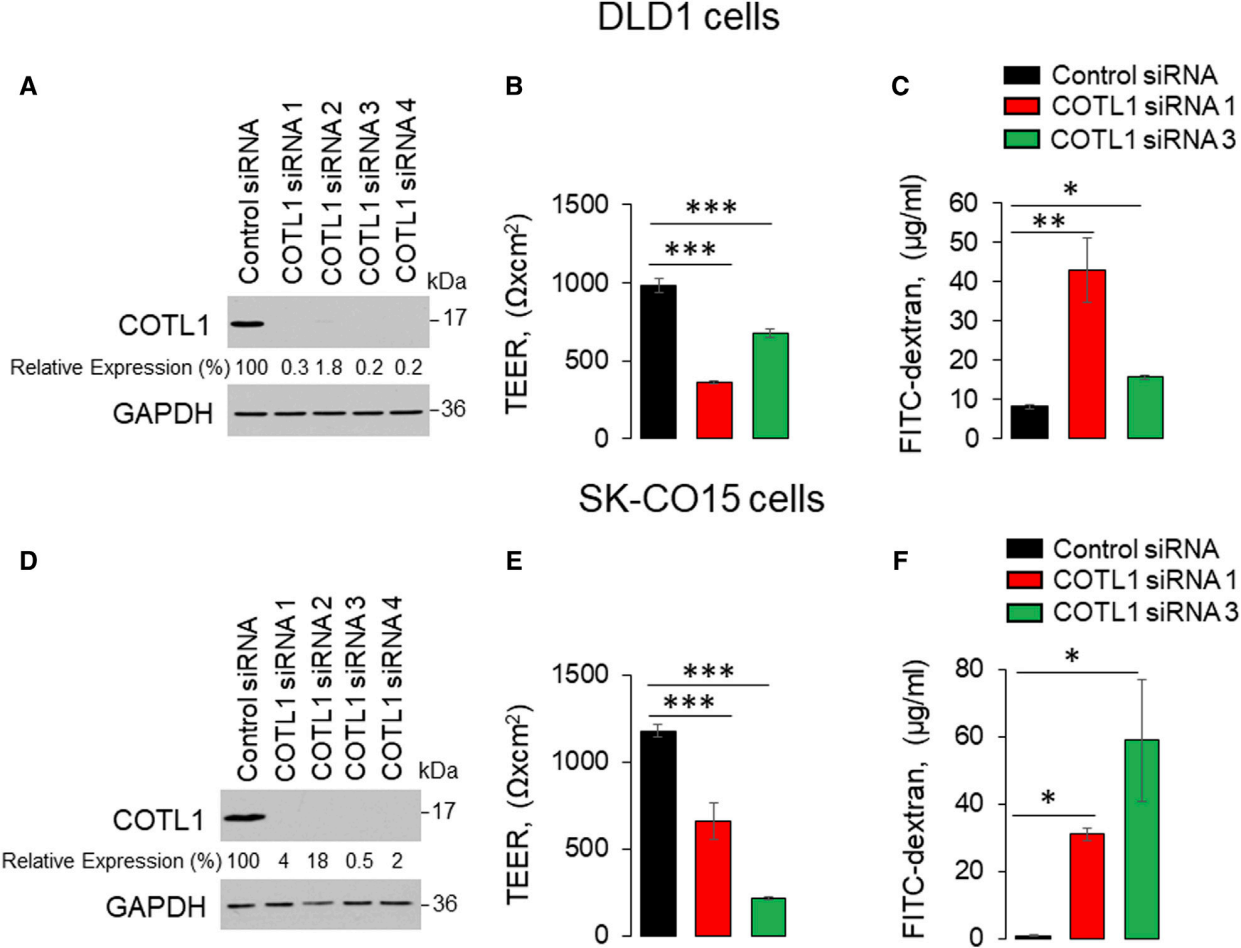
Downregulation of COTL1 increases paracellular permeability of intestinal epithelial cells. **(A, D)** Immunoblotting analysis of COTL1 level in DLD1 **(A)** and SK-CO15 **(D)** cells with siRNA-mediated knockdown of COTL1. **(B, E)** Transepithelial electrical resistance (TEER) of control and COTL1-depleted DLD1 **(B)** and SK-CO15 **(E)** cell monolayers on day 4 after siRNA transfection. **(C, F)** Transmonolayer flux of FITC-dextran in control and COTL1-depleted DLD1 **(C)** and SK-CO15 **(F)** cells on day 4 post siRNA transfection. Data are presented as means ± SE (*n* = 3). *p < 0.05, **p < 0.005, ***p < 0.0005, as compared to control siRNA transfected cells.

To examine the functional role of this actin regulator at epithelial junctions, we used RNA interference to downregulate COTL1 expression in DLD-1 and SK-CO15 cells. These cell lines rapidly establish tight paracellular barriers and were previously used to study the regulation of junctional assembly and remodeling (Hollande et al., 2003; Fujiwara et al., 2015; Lechuga et al., 2015; Cao et al., 2020; Indra et al., 2021; Lechuga et al., 2022). To select efficient siRNAs for the functional studies, IEC were transfected with four different COTL1 siRNA duplexes. Immunoblotting analysis demonstrated that all four siRNAs cause dramatic (up to 99%) downregulation of COTL1 protein expression in both cell lines on day 4 post-siRNA transfection (Figure 2A, D). We used siRNA duplexes 1 and 3 for the subsequent functional studies, as they resulted in a highly-efficient protein depletion (Figure 2A, D) and did not significantly decrease cell viability.

**FIGURE 3:**
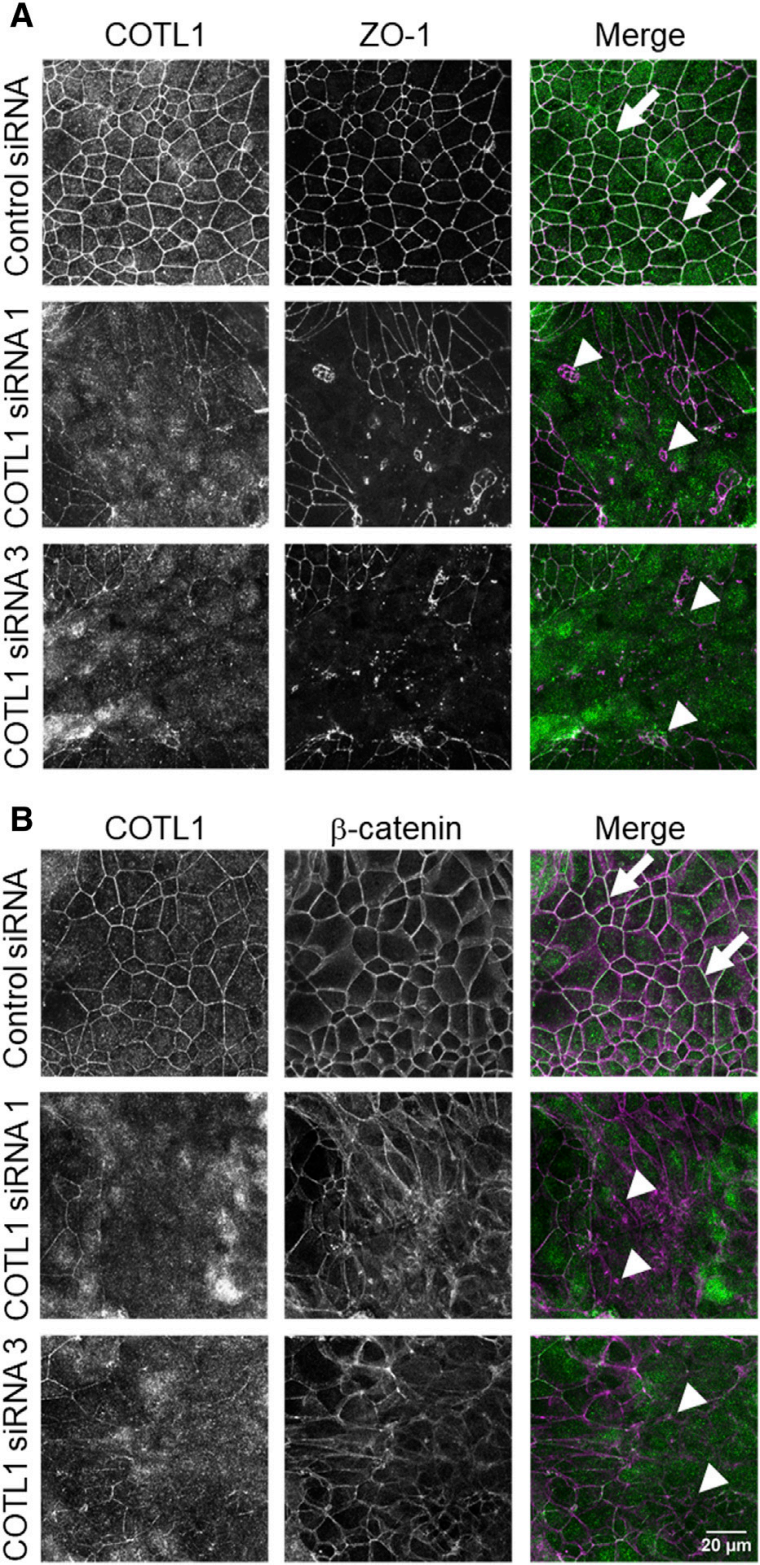
Loss of COTL1 impairs the assembly of epithelial tight and adherens junctions. Confocal microscopy images of control and COTL1-depleted DLD1 cells dual-immunolabeled for COTL1 (green) and either ZO-1 [**(A)**, magenta], or β-catenin [**(B)**, magenta)] on day 4 after siRNA transfection. Arrows indicate intact TJ and AJ in control cell monolayers and arrowheads point at junctional disassembly in COTL1-depleted cells. Images shown are representative of three independent experiments with multiple images taken per slide.

Effects of COTL1 depletion on paracellular permeability were determined by measuring transepithelial electrical resistance (TEER) and transmonolayer flux of FITC-dextran (4 kDa). COTL1 knockdown significantly diminished TEER (Figure 2B, E) and increased FITC-dextran permeability on day 4 post-siRNA transfection (Figure 2C, F) in both DLD1 and SK-CO15 cell lines, thereby indicating disruption of the paracellular barrier. Next, we sought to determine whether this barrier disruption is associated with impaired TJ and/or AJ integrity. Since siRNA depletion of COTL1 appears to be less efficient when performed on Transwell filters, we examined junctional integrity in COTL1-depleted cells cultured on coverslips and specifically focused on the monolayer areas with the most prominent decrease in COTL1 labeling intensity. In control siRNA-treated DLD1 cell monolayers, the TJ and AJ markers, ZO-1, and β-catenin displayed a sharp “chicken wire” labeling pattern that is a characteristic of intact epithelial junctions (Figure 3A, B, arrows). By contrast, this pattern was markedly disrupted in COTL1-depleted DLD1 cells, where junctional localization of ZO-1 was lost and β-catenin was re-localized from the AJs into an intracellular compartment (Figure 3A, B, arrowheads). Similar loss of junctional labeling was observed for E-cadherin, claudin 1, and claudin 4 (Supplementary Figure S3, arrowheads), indicating a general disruption of apical junctional integrity after COTL1 knockdown. Interestingly, confocal *xz* sections revealed formation of more than 1 cell monolayer in COTL1-depleted DLD1 cells (Supplementary Figure S1), which may indicate global alterations in epithelial homeostasis following the loss of this actin-binding protein. Such multilayering was manifested by significantly increased IEC height, from 8.26 ± 0.35 microns in control siRNA-treated cells to 12.34 ± 0.55 and 12.84 ± 0.35 microns in cells treated with COTL1 siRNA 1 and siRNA 3, respectively (*p* < 0.001 as compared to the control group). COTL1 depletion also resulted in the focal disruption of TJs and AJs in SK-CO15 cell monolayers (Supplementary Figure S4, arrowheads) although such junctional disassembly was not as pronounced as in DLD1 cells. Importantly, depletion of COTL1 did not decrease total expression of different TJ and AJ proteins in DLD1 and SK-CO15 cells (Supplementary Figure S5).

Next, we determined whether the described defects in junctional assembly and permeability represent specific consequences of COTL1 depletion, and not caused by the off-target effects of siRNA transfection. Knockdown of COTL1 was performed in DLD1 cells stably expressing either tGFP-tagged COTL1 or control tGFP. COTL1 siRNA dramatically decreased COTL1 levels in tGFP-expressing cells, whereas tGFP-COTL1 cells still retained significant expression of the exogenous protein after siRNA transfection (Supplementary Figure S6A). We found substantial TJ/AJ disruption and significantly increased FITC-dextran permeability in COTL1-depleted DLD1 cells expressing control tGFP, whereas these effects were attenuated in tGFP-COTL1 expressing cells Supplementary Figures S6B, C). This experiment strongly suggests that the observed disruption of epithelial barrier and TJ/AJ disassembly in IEC monolayers represents the specific consequences of COTL1 depletion.

**FIGURE 4:**
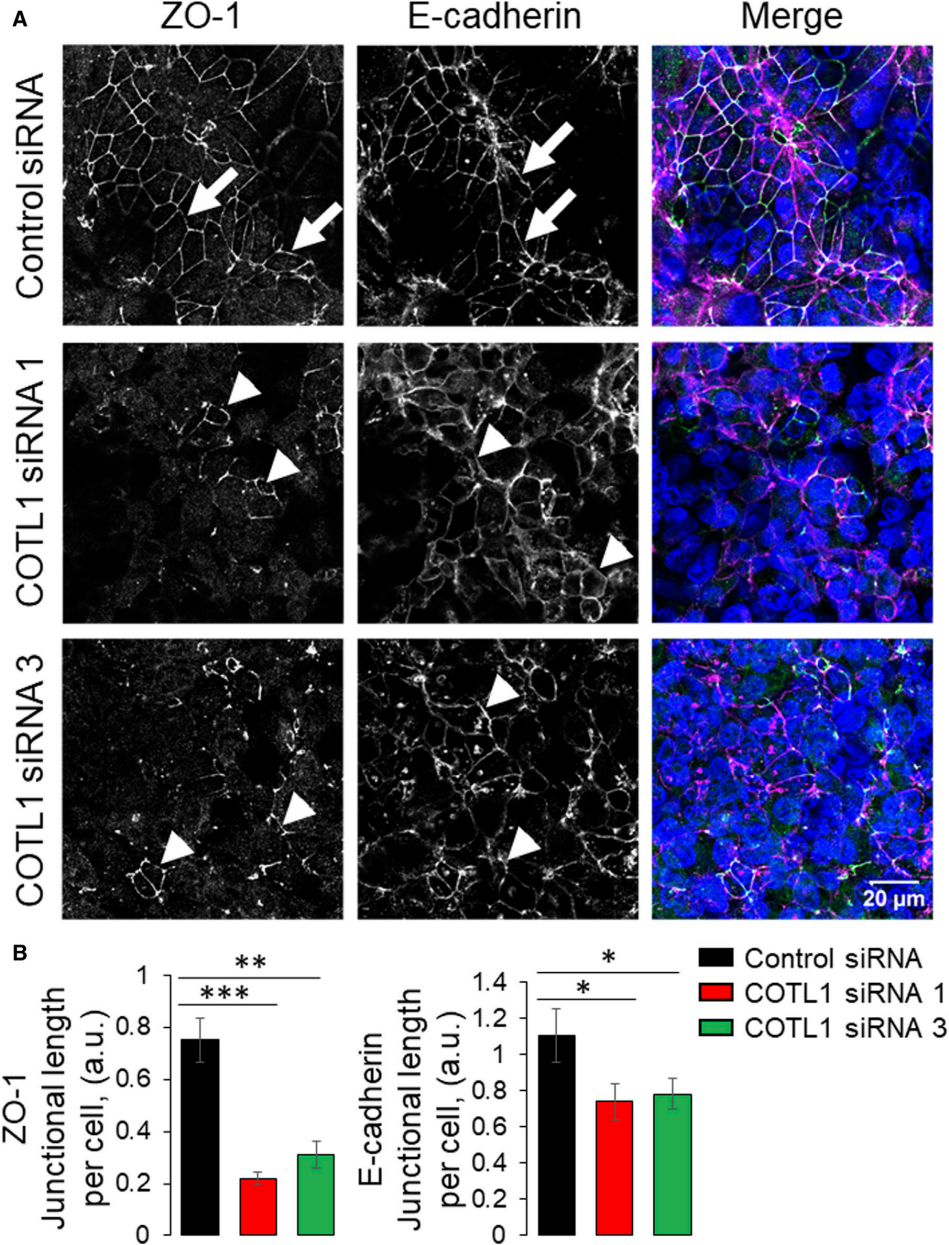
Depletion of COTL1 attenuates AJ/TJ reassembly during the extracellular calcium switch. **(A, B)** Immunolabeling of ZO-1 and E-cadherin in control and COTL1-depleted DLD1 cells after 4 h of extracellular calcium repletion. Representative confocal microscopy images **(A)** and quantification of the normalized junctional length **(B)** are shown. Arrows indicate AJ/TJ reassembly in control cells and arrowheads point at poorly assembled apical junctions in COTL1-depleted cells. Data are presented as means ± SE (*n* = 3). *p < 0.05, **p < 0.005, ***p < 0.0005.

Since the transient COTL1 knockdown was performed in sub-confluent IEC monolayers, different factors including cell proliferation and motility could affect the assembly of apical junctions and establishment of the paracellular barrier. Therefore, we sought to dissect the specific effect of COTL1 depletion on junctional assembly using a calcium switch assay, which is not influenced by cell division and migration (Gonzalez-Mariscal et al., 1990; Ivanov et al., 2005; Lechuga et al., 2015; Lechuga et al., 2022). Confluent control and COTL1-depleted DLD1 cell monolayers were subjected to overnight extracellular calcium depletion to disrupt preexisting junctions, followed by restoration of the normal calcium level to trigger junctional reassembly. Control cells rapidly reassembled AJs and TJs after 4 h of the calcium repletion (Figure 4A, B, arrows). By contrast, the reformation of apical junctions was significantly delayed in COTL1-depleted cells (Figure 4A, B arrowheads). Similar attenuation of calcium-dependent AJ/TJ reassembly was also observed after COTL1 knockdown in SK-CO15 cells (Supplementary Figure S7). Together, these data suggest that COTL1 regulates both the steady-state integrity and reassembly of apical junctions in model IEC monolayers.

### COTL1 depletion disrupts the integrity and reassembly of the perijunctional actomyosin cytoskeleton

**FIGURE 5:**
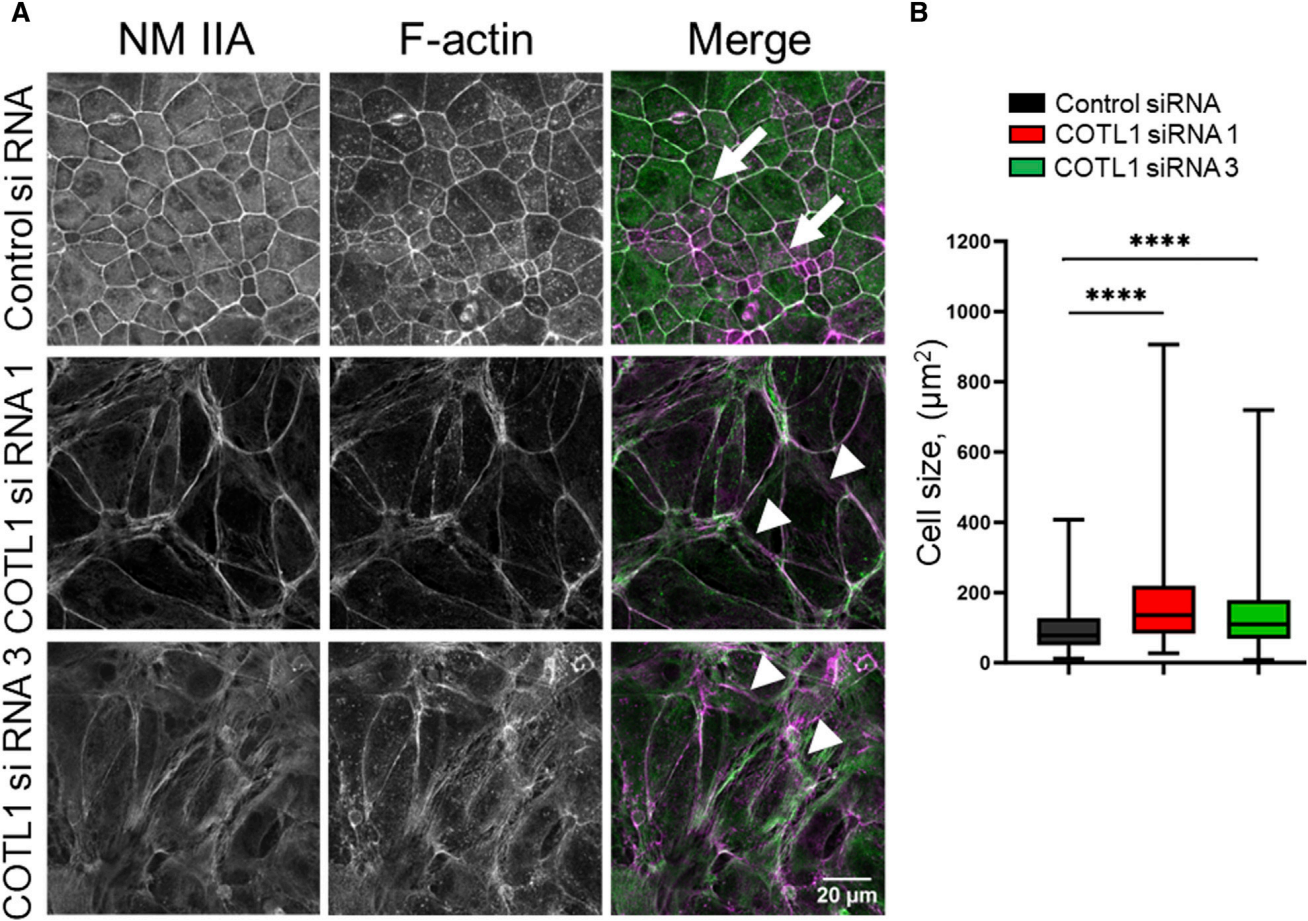
COTL1 depletion impairs organization of the perijunctional actomyosin belt and increases epithelial cell size. **(A)** Confocal microscopy images of control and COTL1-depleted DLD1 cells fluorescently labeled for F-actin (magenta) and a major epithelial myosin II motor, NM IIA (green). Arrows indicate perijunctional actomyosin structures in control IEC. Arrowheads point at disorganization of perijunctional actomyosin bundles in COTL1-depleted IEC. Representative of three independent experiments with multiple images taken per slide. **(B)** Quantification of the cell size of control and COTL1-depleted DLD1 cells. The data are presented as the Box and Whiskers plot with whiskers extending to the minimum and the maximum. ****p < 0.0001; Mann Whitley test.

Next, we sought to determine the mechanisms underlying COTL1-dependent regulation of epithelial apical junctions. Since COTL1 is the F-actin-binding protein and associates with the circumferential F-actin belt in differentiated DLD1 cells (Supplementary Figure S2), one can suggest that it could remodel the perijunctional actomyosin cytoskeleton. The integrity of the actomyosin cytoskeleton was examined by fluorescence labeling for F-actin and NM IIA, which is the most important epithelial NM II paralog previously implicated in the regulation of the intestinal epithelial barrier integrity and repair (Ivanov et al., 2007; Babbin et al., 2009; Naydenov et al., 2016). Control DLD1 cell monolayers displayed a characteristic perijunctional F-actin belt that was enriched with NM IIA (Figure 5A, arrows). This belt was markedly disrupted in COTL1-depleted cells where the tight actomyosin bundles were frequently replaced by disordered and “de-bundled” fibers (Figure 5A, arrowheads). Furthermore, the size of COTL1-depleted DLD1 cells was bigger when compared to the control cells (Figure 5B), which could represent another consequence of their aberrant cytoskeletal organization. We also examined actomyosin reassembly in control and COTL1-depleted IEC during the calcium switch. Control DLD1 cells efficiently reassembled the perijunctional actomyosin belt after 4 h of calcium repletion (Supplementary Figure S8A, arrows). By contrast, the reformation of this cytoskeletal structure was significantly attenuated in COTL1-depleted cells (Supplementary Figure S8A, arrowheads). The delayed reassembly of the junction-associated actomyosin belt was also observed in COTL1-deficient SK-CO15 cells (Supplementary Figure S8B). These data indicate that impaired organization of TJs and AJs in COTL1-depleted cells is associated with abnormal architecture and remodeling of the perijunctional actomyosin cytoskeleton.

### Loss of COTL1 increases permeability of *Drosophila* gut

**FIGURE 6:**
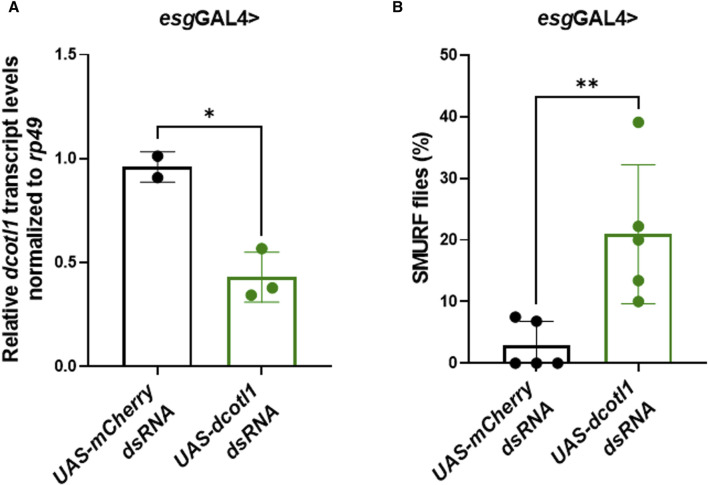
Knockdown of dCOTL1 increases intestinal permeability in aged *Drosophila*. **(A)** qRT-PCR was performed to analyze *dcotl1* transcript levels using RNA harvested from the intestines of 5-day old, female flies expressing *dcotl1* dsRNA or control, *mCherry* dsRNA. Each data point represents 25 intestines. Means ± SD *p < 0.05 **(B)** Smurf assays were performed on 50-day old, female flies expressing *dcotl1* dsRNA or control, *mCherry* dsRNA. % of SMURF positive flies is shown for each genotype. Each data point represents 25 flies. Means ± SD; **p < 0.01. Mann Whitney test.

To determine whether COTL1 knockdown could impair the function of the intestinal epithelial barrier *in vivo*, dsRNA targeting the *Drosophila melanogaster* COTL1 homolog, dCOTL1, was expressed in adult intestinal stem cells under the control of *esg*GAL4. qRT-PCR analyses of 5-day old flies demonstrated approximately 50% knockdown of *dcotl1* gene expression (Figure 6A). SMURF assays performed on 50-day old female flies demonstrated that *dcotl1* dsRNA expressing flies exhibited significantly elevated percentages of SMURFs, as compared to *GFP* dsRNA expressing female controls (Figure 6B). These data suggest that, similar to results observed in human cells, dCOTL1 knockdown disrupts the integrity of intestinal epithelial barrier, *in vivo*, in *Drosophila*.

### COTL1 depletion inhibits collective IEC migration

**FIGURE 7:**
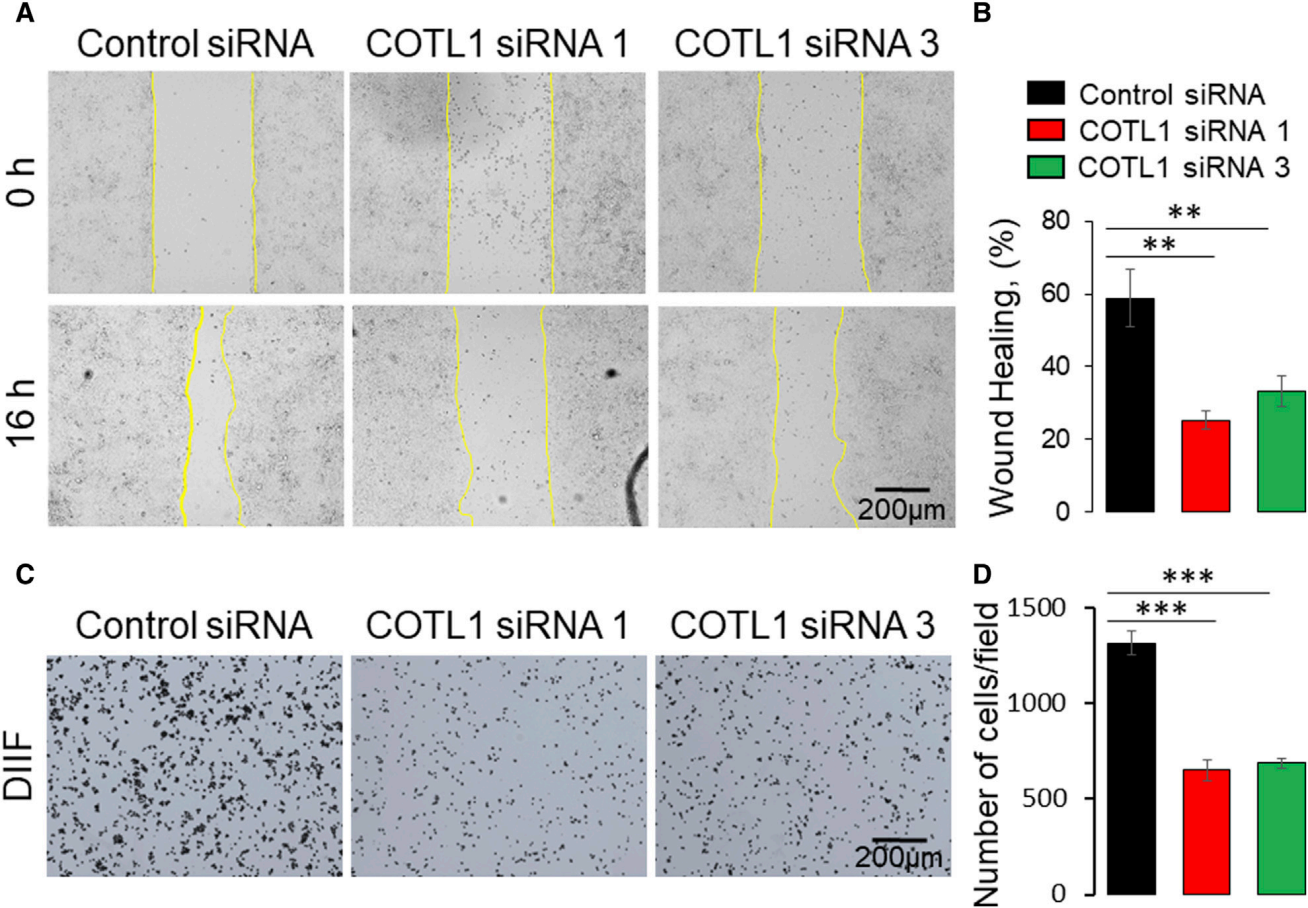
Depletion of COTL1 inhibits collective epithelial cell migration and cell-matrix attachment. **(A, B)** Wound healing assay in control and COTL1-depleted DLD1 cells. Representative wound images **(A)** and quantification of the wounded area **(B)** are shown. **(C, D)** Collagen I matrix attachment assay of control and COTL1-depleted DLD1 cells. Representative images of adhered cells **(C)** and quantification of cells adhered at 30 min after plating **(D)**. Mean ± SE [**(A, B)**: *n* = 4; **(C, D)**: *n* = 3]; **p < 0.01; ***p < 0.0005, as compared to the control siRNA group.

Apical junctions play critical roles in regulating epithelial wound repair by orchestrating collective cell migration and matrix deposition in injured epithelial layers (Ozawa et al., 2020; Hadjisavva et al., 2022; Tholmann et al., 2022). Given the observed roles of COTL1 in promoting junctional assembly, we sought to examine if this protein also controls IEC motility. Immunofluorescence labeling and confocal microscopy observed a specific enrichment of COTL1 at the membrane protrusions and actin bundles at the migrating edge of wounded DLD1 cell monolayers (Supplementary Figure S9A, arrow). Furthermore, loss of COTL1 affected F-actin organization at the migrating cell edge by causing a disappearance and de-bundling of F-actin arcs that stabilize the migrating cell front (Supplementary Figure S9B, arrowheads). Importantly, depletion of COTL1 significantly attenuated the collective migration of DLD1 (Figure 7A, B) and SK-CO15 cell monolayers (Supplementary Figure S10A, B) in the wound healing assay. By contrast, loss of this protein resulted in a significant acceleration of transfilter migration of individual SK-CO15 in the Boyden Chamber (Supplementary Figure S10C). However, it should be noted that we were unable to induce significant DLD1 cell migration in the Boyden Chambers. The opposite effects of COTL1 on the collective and the individual IEC migration suggest that dysregulation of epithelial junctions could contribute to attenuated wound healing in COTL1-depleted epithelial monolayers. We also examined IEC attachment to the extracellular matrix (ECM), which is an essential step for epithelial cell motility. COTL1-depletion significantly decreased the attachment of DLD1 (Figure 7C, D) and SK-CO15 cells (Supplementary Figure S10E, F) to collagen I. Together, our findings suggest that COTL1 serves as a positive regulator in the collective migration and ECM attachment of IEC monolayers.

### Overexpression of COTL1 enhances integrity of the model IEC barrier

**FIGURE 8:**
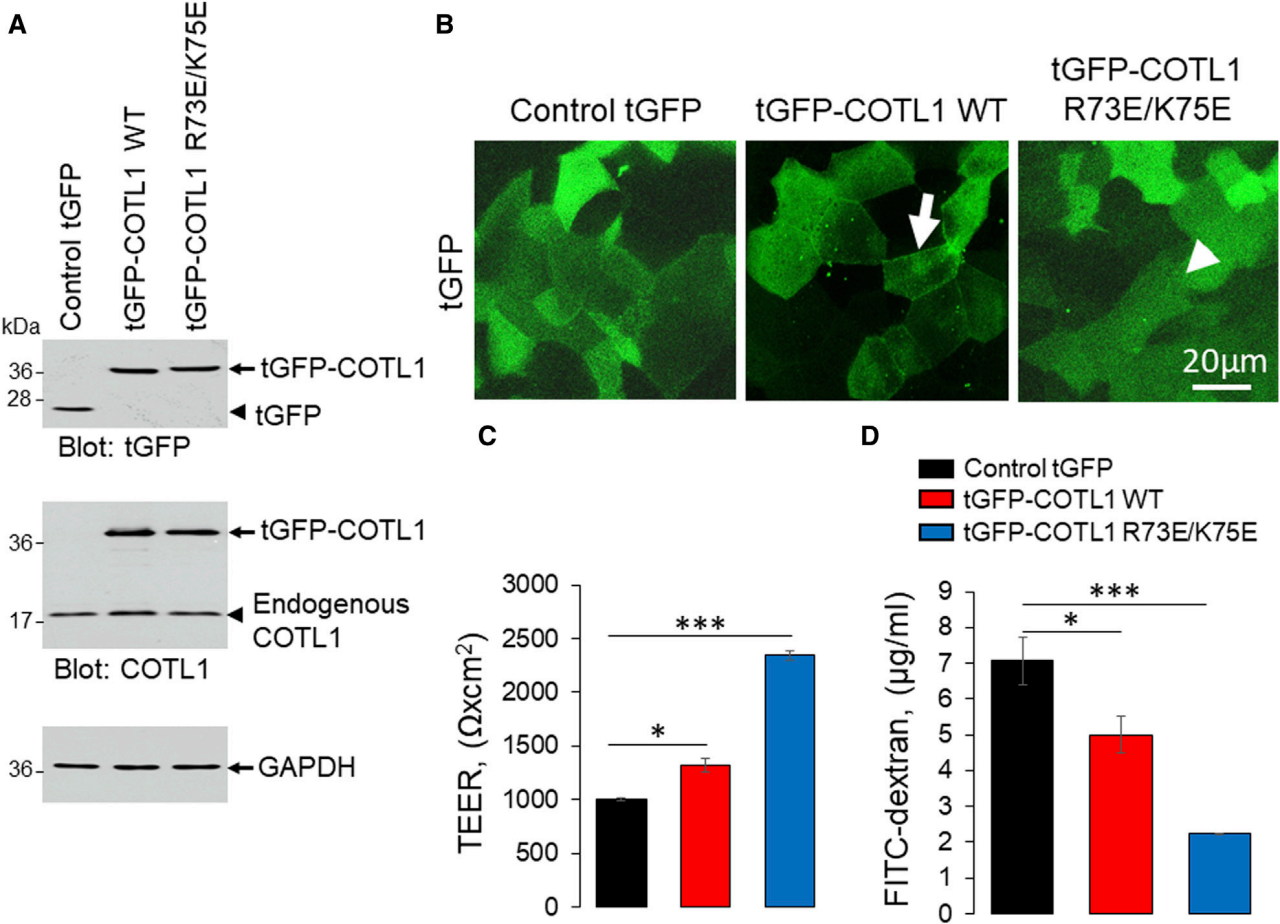
Overexpression of wild type COTL1 and its F-actin binding deficient mutant decreases IEC barrier permeability. **(A)** Immunoblotting analysis of DLD1 cells expressing either control tGFP, tGFP-tagged full-length COTL1 (tGFP-COTL1 WT) or the COTL1 mutant unable to bind F-actin (tGFP-COTL1 R73E/K75E). **(B)** Imaging of tGFP fluorescence in DLD1 cells overexpressing either control tGFP, tGFP-COTL1 WT, or tGFP-COTL1 R73E/K75E. Arrow indicates localization of wild-type tGFP-COTL1 at epithelial junctions. Arrowhead points at non-junctional localization of the COTL1 actin-uncouple mutant. **(C)** Transepithelial electrical resistance and **(D)** transmonolayer FITC-dextran flux in DLD1 cells expressing control tGFP, tGFP-COTL1 WT or tGFP-COTL1 R73E/K75E mutant. Means ± SE (*n* = 3); *p < 0.05, ***p < 0.0005 as compared to control tGFP expressing cells.

**FIGURE 9:**
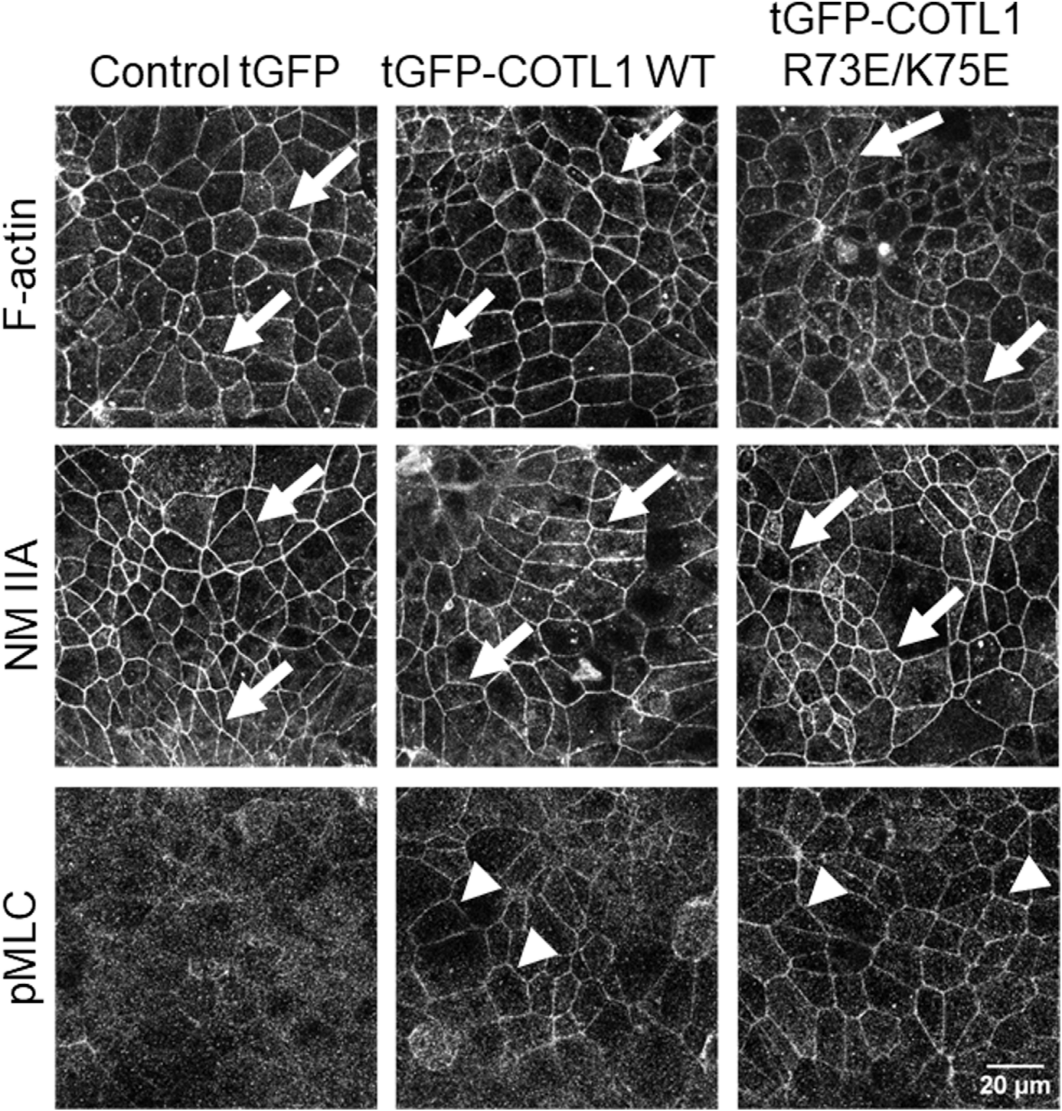
Overexpression of wild type COTL1 and its F-actin binding deficient mutant increases phosphorylation of junction-associated NM II.

Given the known interactions of COTL1 with actin filaments (de Hostos et al., 1993; Provost et al., 2001a; Dai et al., 2006) and the observed abnormal assembly of the perijunctional F-actin belt in COTL1 depleted IEC (Figure 5; Supplementary Figure S8), we rationalized that the regulatory effects of COTL1 on the IEC barrier integrity and repair could depend on its direct interactions with actin filaments. To test this hypothesis, we generated DLD1 cell lines stably expressing tGFP-labeled wild-type COTL1, a COTL1 R73E/K75E mutant that does not bind actin (Kim et al., 2014; Liu et al., 2018), or control tGFP. The exogenous wild type and mutant COTL1 were expressed at similar levels, about 2-fold and 1.7-fold higher than the endogenous COTL1 expression (Figure 8A). Interestingly, while wild-type tGFP-COTL1 significantly accumulated at apical junctions (Figure 8B, arrow), no junctional enrichment was characteristic for the actin-uncoupled COTL1 mutant (Figure 8B, arrowhead). Overexpression of WT COTL1 resulted in tightening of the paracellular barrier according to the increased TEER (Figure 8C) and decreased transepithelial FITC-dextran flux (Figure 8D). Surprisingly, such barrier-enhancing effects were even more pronounced in the cells overexpressing the actin uncoupled COTL1 mutant (Figures 8C, D). We further examined the effects of COTL1 overexpression on the IEC barrier by visualizing apical junctions. While no marked alterations in the TJ and AJ assembly were detected (Supplementary Figure S11A), immunofluorescence labeling and confocal microscopy revealed ZO-1 accumulation at the multicellular and tricellular vertices in COTL1 mutant overexpressing DLD1 cells (Supplementary Figure S11A, B arrows). Furthermore, overexpression of either wild type or the actin filament uncoupled COTL1 mutant did not markedly affect the integrity of the perijunctional actomyosin belt (Figure 9, arrows). Overexpression of both WT and mutant COTL1, however, increased MLC phosphorylation at the perijunctional F-actin belt (Figure 9, arrowheads), which is indicative of increased activity of junction-associated NM II. Thus, the relative signal intensity of perijunctional pMLC was 2.26 ± 0.19 and 2.23 ± 0.28 fold higher in DLD1 cells expressing the wild type and mutant COTL1, respectively, comparing to control tGFP-expressing cells. Immunoblotting analysis revealed no effects of COTL1 overexpression on total pMLC level and no changes in other cytoskeleton-related signaling, such as phosphorylation of cofilin and ezrin-moesin-radixin (ERM) proteins (Supplementary Figure S12). Together, the described data suggest that while COTL1 enhances the IEC barrier its barrier-enhancing activity is uncoupled from the actin filament binding.

### Overexpression of the actin uncoupled COTL1 mutant inhibits IEC migration and matrix attachment

**FIGURE 10:**
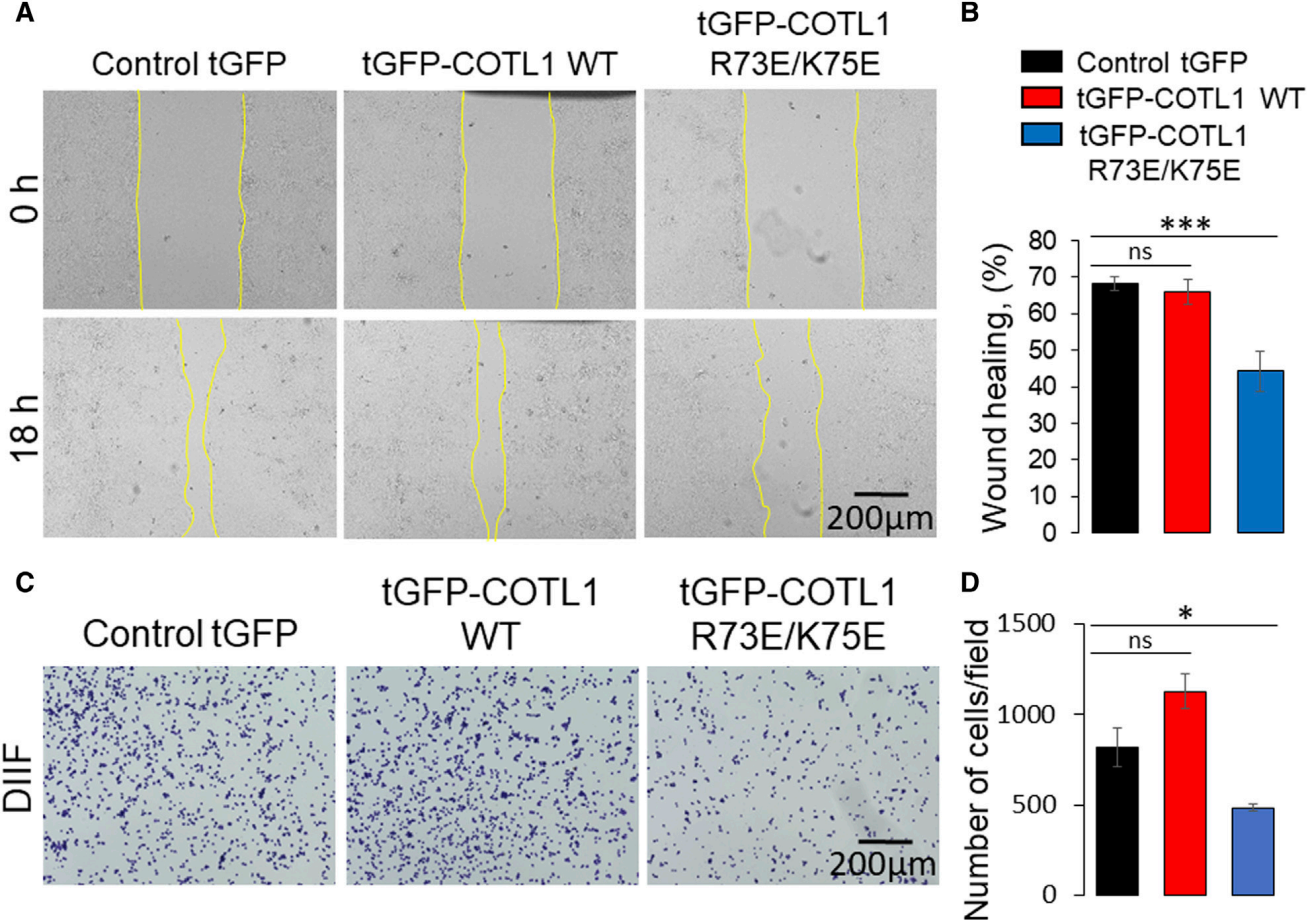
Expression of the F-actin binding deficient COTL1 mutant inhibits collective migration and matrix attachment of IEC. **(A, B)** Wound closure of DLD1 cell monolayers stably expressing control tGFP, tGFP-COTL1 WT or tGFP-COTL1 R73E/K75E. Representative images **(A)** and quantification of the wound area **(B)** are shown. **(C, D)** Collagen I matrix attachment assay of DLD1 cells stably expressing control tGFP, tGFP-COTL1 WT or tGFP-COTL1 R73E/K75E. Representative images of adhered cells **(C)** and quantification of cells adhered at 30 min after plating **(D)**. Mean ± SE [**(A, B)**: *n* = 4; **(C, D)**: *n* = 3]. *p < 0.05, ***p < 0.0005 as compared to control GFP expressing cells.

Given the unexpected barrier-enhancing effect of the R73E/K75E COTL1 mutant, we next sought to determine whether this mutant affects IEC motility. To do this, we compared the behavior of wild-type tGFP-COTL1 and its actin uncoupled mutant in wounded DLD1 cell monolayers using live cell imaging. COTL1 WT was dynamically recruited to the membrane protrusions and stress fibers at the migrating cell edge (Supplementary Movie S1), thereby recapitulating the localization of endogenous COTL1 (Supplementary Figure S9). By contrast, the actin uncoupled COTL1 mutant did not accumulate at membrane protrusions or fibrillar structures at the migrating cell edge (Supplementary Movie S1). Furthermore, overexpression of the actin uncoupled COTL1 mutant significantly delayed collective DLD1 cell migration (Figures 10A, B) and inhibited cell-ECM attachment (Figures 10C, D), whereas overexpression of WT COTL1 did not affect these processes (Figure 10). Finally, overexpression of the actin uncoupled COTL1 mutant impaired actin-filament assembly at the migrating cell edge (Supplementary Figure S13, arrowheads), recapitulating the effects of COTL1 depletion. It is noteworthy that we also tested the cellular effects of the COTL1 K131A mutant that is unable to interact with 5-LO (Provost et al., 2001b). However, stable expression of the 5-LO-uncoupled mutant had the same functional consequences as expression of exogenous WT COTL1 in DLD1 cells (Supplementary Figure S14). Thus, similar to WT COTL1, expression of the COTL1 K131A mutant modestly increased TEER, decreased FITC-dextran flux, and did not affect wound closure (Supplementary Figures S14B–D). This indicates that COTL1 regulates IEC barrier integrity and repair via mechanisms that are independent of 5-LO binding.

## Discussion

Epithelial AJs and TJs are linked to the cortical actomyosin cytoskeleton, which plays critical roles in regulating junctional structure and function (Mege and Ishiyama, 2017; Charras and Yap, 2018; Citi, 2019; Varadarajan et al., 2019). A number of different actin-binding proteins associate with epithelial junctions to facilitate AJ/TJ assembly and connection to the perijunctional actomyosin cytoskeleton (Van Itallie et al., 2013; Van Itallie et al., 2014; Tan et al., 2020; Suarez-Artiles et al., 2022). The present study identifies COTL1 as a novel component of the junction-associated actomyosin cytoskeleton that is important for epithelial barrier integrity and dynamics. Furthermore, our study highlights COTL1 as a positive regulator of collective migration of IEC, which could play an important role in the repair of injured epithelial barriers.

We found the enrichment of endogenous COTL1 at apical junctions in several well-differentiated human colonic epithelial cell lines (Figure 1; Supplementary Figure S1). Likewise, exogenously expressed COTL1 accumulated at apical junctions (Figure 8B). Such junctional recruitment of COTL1 was found to be dependent on its interactions with actin filaments since depolymerization of actin filaments impaired COTL1 localization at epithelial cell-cell contacts (Supplementary Figure S2A) and the COTL1 mutant unable to bind actin did not show specific junctional accumulation (Figure 8B). Most importantly, our data suggest that COTL1 represents a previously unrecognized positive regulator of epithelial barrier permeability and junctional integrity. The barrier-enhancing activity of this protein is supported by our loss-of-function and gain-of-function data (Figures 2, 8). Furthermore, we described the essential roles of COTL1 in controlling the steady-state TJ and AJ integrity in confluent IEC monolayers (Figure 3; Supplementary Figures S3, S4) as well as the orchestrated junctional reassembly during the extracellular calcium switch (Figure 4; Supplementary Figure S7). While no previous studies have reported the roles of COTL1 in regulating epithelial or endothelial junctions, COTL1 was previously shown to accumulate at the T-cells - B-cells contact site and control the F-actin dynamics in this immunological synapse (Kim et al., 2014). It is tempting to speculate, therefore, that COTL1 could serve as an important regulator of intercellular adhesions in different cell types. Our data demonstrating COTL1-dependent regulation of *Drosophila* gut barrier permeability (Figure 6) suggest that this protein could also control the assembly and integrity of tissue barriers *in vivo*. Future studies involving mice models with tissue-specific knockout of COTL1 are needed to better characterize the physiological roles of this actin-binding protein.

What are the mechanisms of COTL1-dependent regulation of epithelial apical junctions? Our results demonstrate that impaired assembly of epithelial junctions in COTL1-depleted IEC monolayers is associated with an abnormal organization and remodeling of the perijunctional actomyosin cytoskeleton (Figure 5; Supplementary Figure S8). COTL1 appears to regulate the architecture of the circumferential actomyosin belt that was transformed into a loosely packed actomyosin filament network following COTL1 knockdown (Figure 5). Interestingly, calcium repletion experiment suggests that COTL1 is recruited to the perijunctional cytoskeleton after the assembly of the circumferential F-actin belt (Supplementary Figure S2B). This indicates that COTL1 is dispensable for the *de novo* formation of the perijunctional actin filaments, and could control their assembly into the tight circumferential belt. The exact mechanisms underlying the cytoskeletal abnormalities in COTL1 depleted IEC remain to be elucidated, but they could involve several steps of the complex regulation of the actin filament dynamics. It is possible that COTL1 regulates stability of actin filaments by competing with other ADF-H domain proteins. Furthermore, it can control NM II dependent cross-linking of actin filaments, or transduction of mechanical forces at the cell cortex, given our data about COTL1 dependent regulation of MLC phosphorylation (Figure 9). Finally, COTL1 cold modulate actin filament interactions with other cross-linking proteins.

Given the key roles of the cortical actomyosin filaments in apical junction structure and remodeling, modulation of the perijunctional cytoskeleton is likely to underline the effects of COTL1 on IEC barrier and junctions. This suggestion is in line with the actin cytoskeleton-dependent control of epithelial junctions and cell polarity previously reported for other ADF-H domain homology proteins, cofilin-1 and drebrin (Bazellieres et al., 2012; Wang et al., 2016). However, our results argue that the effects of COTL1 on the IEC barrier and junctions could be independent of its direct binding to actin filaments. Surprisingly, the actin-binding deficient COTL1 mutant, which is not recruited to apical junctions, displayed superior barrier-enhancing effects as compared to the WT protein (Figure 8). This paradoxical result suggests that COTL1 is likely to regulate IEC barrier indirectly, by affecting cellular signaling. This idea is in agreement with previous studies implicating COTL1 in the control of signaling events that target the cytoskeleton. For example, the knockdown of COTL1 decreased cofilin phosphorylation in chicken neurons (Hou et al., 2021), whereas COTL1 overexpression attenuated MAPK/ERK phosphorylation in breast cancer cells (Xia et al., 2018). While we did not observe significant effects of COTL1 overexpression on total cofilin, ERM, or MLC phosphorylation (Supplementary Figure S12), expression of barrier enhancing WT COTL1 or its R73E/K75E mutant increased the activatory phosphorylation of MLC at the perijunctional actin belt (Figure 9). Such findings indicate that COTL1 modulates local cytoskeletal signaling at epithelial junctions. While the exact nature of such signaling regulation remains to be investigated, it fits into the current concept of the mechanosensitive control of epithelial junctions. The key part of this concept postulates the existence of local signaling feedback loops formed by the interplay between NM II assembly and recruitment of RhoA small GTPase and its negative and positive regulators (Priya et al., 2015; Priya et al., 2017). Thus, assembly of thick perijunctional actomyosin bundles create stable zones of active junction associated RhoA by inhibiting cortical localization of negative regulators of Rho activity. Such RhoA activation, in turn, promotes NM II phosphorylation and contractility (Wu and Priya, 2019). We speculate that COTL1 could serve as a part of these cortical signaling feedback loops by sequestering and locally inactivating negative regulators of RhoA activity. The actin-binding-deficient COTL1 mutant that does not accumulate at junctions has bigger barrier-protective effects because it may sequester these negative regulators away from epithelial junctions. There are precedents for the regulation of junctional RhoA signaling by actin-scaffolding proteins. For example, cortactin, which is an essential driver of actin polymerization, was shown to inactivate cortical RhoA in intestinal epithelial cells (Citalan-Madrid et al., 2017; Liang et al., 2017). While the molecular identity of negative signaling regulators that could be sequestered by COTL1 remains unknown, different Rho GAPs could be possible candidates for future studies.

Our data suggest that in addition to controlling the integrity of model IEC barriers, COTL1 serves as a positive regulator of collective cell migration, which could be essential for the repair of injured epithelial layers. Both endogenous (Supplementary Figure S9A) and exogenously expressed COTL1 (Supplementary Movie S1) accumulated in membrane protrusions and cytoskeletal structures at the migrating wound edge, whereas the actin binding-deficient COTL1 mutant did not show such localization (Supplementary Movie S1). Depletion of COTL1 and overexpression of its actin-uncoupled mutant inhibited IEC wound healing (Figures 7, 10) and disrupted the F-actin architecture at the migrating wound edge (Supplementary Figures S9B, S13), suggesting that COTL1 stimulates collective cell migration via an actin-binding dependent mechanism. These findings are consistent with previous studies conducted in other experimental systems. For example, loss of COTL1 expression inhibited migration and invasion of lung cancer cells, whereas COTL1 overexpression promoted lung cancer cell motility (Guo et al., 2017). Furthermore, COTL1 depletion inhibited neuronal growth cone extension and neural crest cell migration (Hou et al., 2013; Hou et al., 2021). Importantly, expression of the COTL1 mutants unable to interact with actin filaments inhibited neural crest cell migration (Hou et al., 2013) and attenuated T-cell spreading (Kim et al., 2014), recapitulating the functional effects of COTL1 knockdown in these experimental systems. Together, our results along with previously published studies delineate two different mechanisms underlying COTL1-dependent regulation of cell adhesion and motility. The first mechanism that is essential for cell migration requires COTL1 binding to actin filaments, which likely alters the filament stability and turnover. The second essential mechanism is that the regulation of epithelial junctions does not require direct COTL1 binding to actin filaments and involves signaling events leading to modulation of NM II activity.

Given the important roles of COTL1 in controlling intercellular adhesions and cell migration, one could suggest that dysregulation of this protein contributes to human diseases. The disease relevance of COTL1, however currently remains poorly understood. COTL1 was found to be markedly upregulated in lung cancer (Jeong et al., 2011; Guo et al., 2017), breast cancer (Pei et al., 2020; Wang et al., 2022) and glioblastoma (Shao et al., 2020) but was downregulated in liver metastases of colon cancer (Kim et al., 2019). Importantly, high COTL1 expression was shown to correlate with poor disease prognosis for breast cancer and glioblastoma patients (Shao et al., 2020; Wang et al., 2022), which suggests this actin-binding protein plays a causal role in tumor progression. In addition, some evidence associates COTL1 with immune disorders. For example, COTL1 expression was found to be upregulated in rheumatoid arthritis patients and its genetic polymorphism was associated with higher susceptibility to this autoimmune disease (Jin et al., 2009). Furthermore, a recent study identified COTL1 among genes that were differentially expressed in Crohn’s disease patients who are resistant to the anti-tumor necrosis factor therapy (Kwak et al., 2023). Despite emerging associations of COTL1 with different human diseases, little is known about the regulation of its expression and function in disease states. One interesting regulatory mechanism involves the de-repression of COTL1 expression by genetic silencing of different microRNA species, such as microRNA-30c-5p in breast cancer (Pei et al., 2020) and microRNA-506-3p in lung cancer (Guo et al., 2017).

In conclusion, the present study identifies COTL1 as a novel regulator of the IEC barrier integrity and repair. COTL1 enhances barrier integrity by promoting TJ and AJ assembly and regulating establishment of the perijunctional actomyosin cytoskeleton. It accelerates barrier repair by promoting collective migration and matrix adhesion of IEC. Furthermore, our study emphasizes multiple mechanisms that underline the described effects of COLT1 on epithelial barrier integrity and cell migration. These mechanisms could be either dependent or independent of COTL1 binding to actin filaments. The described functional activities of COTL1 suggest that this poorly studied cytoskeletal protein could play important roles in regulating tumor metastasis and disruption of epithelial barriers in tissue inflammation.

## Data Availability

The raw data supporting the conclusions of this article will be made available by the authors, without undue reservation.

## Abbreviations

ADF, actin-depolymerizing factor; AJs, adherens junctions; COTL1 coactosin-like protein 1; ECM, extracellular matrix; IEC, intestinal epithelial cells; 5-LO, 5-lipooxygenase; MLC, myosin light chain; NM II, non-muscle myosin II; TJs, tight junctions; ZO, zonula occludens.
