# Supplementary material for: Coactosin-like protein 1 regulates integrity and repair of model intestinal epithelial barriers via actin binding dependent and independent mechanisms
Source: Front Cell Dev Biol. 2024 Jul 8;12:1405454. doi: 10.3389/fcell.2024.1405454 (PMC11260685; doi:10.3389/fcell.2024.1405454)
Supplement: Supplementary file 2 [file Table1.DOCX]

**Supplementary Figure Legends:**

**Supplementary Figure 1. COTL1 is selectively enriched at apical junctions in polarized colonic epithelial cells.** xz projections of dual-immunofluorescence images of COTL1 (green) and either ZO-1 (**A**, magenta) or beta-catenin (**B**, magenta) in control and COTL1 depleted DLD1 cells. Arrows point at COTL1 accumulation at epithelial apical junctions which disappears after COTL1 knockdown.

**Supplementary Figure 2. COTL1 shows a dynamic accumulation at the perijunctional F-actin belt.** (**A**) Dual-fluorescence labeling of COTL1 (green) and F-actin (magenta) in control polarized DLD1 cell monolayers and DLD1 cell monolayers treated for 1 h with Latrunculin B (1 μM). Arrow points at COTL1 enrichment at the perijunctional F-actin belt in control cell monolayers. Arrowhead points at disruption of junctional COTL1 labeling after disassembly of the perijunctional actin cytoskeleton. (**B**) Fluorescence labeling of COTL1 and F-actin in DLD1 cells after overnight extracellular calcium depletion and at different times of calcium repletion. Arrows point at the reassembly of the perijunctional F-actin belt and junctional accumulation of COTL1 during calcium repletion.

**Supplementary Figure 3. Downregulation of COTL1 impairs junctional localization of different TJ and AJ proteins.**

Confocal microscopy images of control and COTL1-depleted DLD1 cells immunolabeled for E-cadherin, claudin 1, and claudin 4 on day 4 after siRNA transfection. Arrows indicate intact TJs and AJs in control cell monolayers and arrowheads point at junctional disassembly in COTL1-depleted epithelial cells.

**Supplementary Figure 4.** **Downregulation of COTL1 induces focal disruption of epithelial TJ and AJ integrity in steady-state SK-CO15 cell monolayers**.

Confocal microscopy images of control and COTL1-depleted DLD1 cells immunolabeled for ZO-1 (green) and β-catenin (magenta). Arrows indicate intact TJs and AJs in the control epithelial cell monolayer, whereas arrowheads point at the focal junctional disassembly in COTL1-depleted SK-CO15 cells.

**Supplementary Figure 5. Knockdown of COTL1 does not affect expression of different TJ and AJ proteins.**

Immunoblotting analysis of different junctional protein expression in control and COTL1 depleted DLD1 (**A**) and SK-CO15 (**B**) cells on day 4 after siRNA transfection. Representative immunoblots of two independent experiments are shown.

**Supplementary Figure 6. COTL1 overexpression rescues junctional disassembly and IEC barrier disruption caused by COTL1 knockdown.**

Parental DLD1 cells and cells stably expressing either control tGFP or wild-type tGFP-COTL1 were treated with either control or COTL1-specific siRNAs 1. (**A**) Immunoblotting analysis showing expression of endogenous COTL1 and exogenous tGFP-COTL1 in COTL1 depleted and rescued DLD1 cells on day 4 after siRNA transfection. (**B**) Immunofluorescence labeling of ZO-1 and E-cadherin in control tGFP and tGFP- COTL1- expressing cells transfected with either control or COTL1 siRNAs on day 4 of the knockdown. Arrowheads point at disrupted TJs and AJs in tGFP-expressing, COTL1-depleted cells. Arrows point at intact junctions in tGFP-COTL1 rescued, COTL1-depleted cells. (**C**) Transmonolayer FITC-dextran flux in control tGFP and tGFP-COTL1 expressing cells transfected with either control or COTL1 siRNA on day 4 of the knockdown. Mean ± SE (n =3). **p< 0.005 as compared to control siRNA transfected, control tGFP expressing cells.

**Supplementary Figure 7. Depletion of COTL1 attenuates AJ/TJ reassembly in SK-CO15 cells subjected to the calcium switch.**

(**A,B**) Immunolabeling of ZO-1 (green) and E-cadherin (magenta) in control and COTL1-depleted SK-CO15 cells after 3 h of extracellular calcium repletion. Representative confocal microscopy images (**A**) and quantification of the normalized junctional length (**B**) are shown. Arrows indicate AJ/TJ reassembly in control cells and arrowheads point at poorly assembled apical junctions in COTL1-depleted cells. Means ± SE (n=3). **p< 0.005 as compared to control siRNA transfected cells.

**Supplementary Figure 8.** **Depletion of COTL1 attenuates reassembly of the perijunctional actomyosin cytoskeleton.** Fluorescence labeling of F-actin and NM IIA in control and COTL1 depleted DLD1 (**A**) and SK-CO15 (**B**) cells after 4 h and 3 h of extracellular calcium repletion, respectively. Arrows indicate actomyosin reassembly in control cells and arrowheads point at poorly actomyosin reassembled in COTL1-depleted epithelial cells. Representative of two independent experiments with multiple images taken per slide.

**Supplementary Figure 9. COTL1 localizes in membrane protrusions and its depletion disorganizes the actin cytoskeleton at the migrating IEC edge.**

(**A**) Dual fluorescence labeling of COTL1 (green) and F-actin (magenta) at the migrating edge of wounded DLD1 cells. Arrows point at COTL1 colocalization with F-actin bundles in membrane protrusions. (**B**) Fluorescence labeling of F-actin at the migrating edge of wounded control and COTL1-depleted DLD1 cells. Arrow indicates prominent F-actin arcs in control IEC. Arrowheads point at de-bundled actin filaments in COTL1-depleted cells.

**Supplementary Figure 10.** **Loss of COTL1 has variable effects on SK-CO15 cell migration and inhibits cell-matrix attachment.**

(**A,B**) Wound healing assay in control and COTL1-depleted SK-CO15 cell monolayers. Representative wound images (**A**) and quantification of wound closure (**B**) are shown.

(**C,D**) Boyden Chamber migration assay of control and COTL1-depleted SK-CO15 cell monolayers. Representative images of migrated cells (**C**) and quantification of transmigrated cells (**D**) are shown. (**E,F**) Collagen I matrix attachment assay of control and COTL1-depleted SK-CO15 cell monolayers. Representative images of migrated cells (**E**) and quantification of cells adhered after 20 min incubation (**F**) are shown. Mean ± SE (**A,B**: n =4; **C-F**: n=3); *p< 0.05, **p< 0.005, ***p< 0.0005 as compared to the control siRNA group.

**Supplementary Figure 11. Overexpression of the F-actin binding deficient COTL1 mutant increases ZO-1 accumulation at multicellular junctions.**

(**A**) Confocal microscopy images of DLD1 cell monolayers stable expressing control tGFP, tGFP-COTL1 WT, or tGFP-COTL1 R73E/K75E. Cells are immunofluorescence labeled for E-cadherin and ZO-1. Arrows point at ZO-1 accumulation in multicellular junctions of tGFP-COTL1 R73E/K75E expressing cells. (**B**) Calculated ratio of ZO-1 intensity in multicellular versus bicellular junctions. Mean ± SE (n=3); *p< 0.05 as compared to control tGFP expressing cells. Representative of three independent experiments with multiple images taken per slide.

**Supplementary Figure 12. Overexpression COTL1 does not affect global actin cytoskeleton-targeting signaling.**

Immunoblotting analysis of the expression of total and phosphorylated (p) forms of MLC, cofilin, and ERM protein expression in DLD1 cell monolayers stable expressing control tGFP, tGFP-COTL1 WT or tGFP-COTL1 R73E/K75E. Representative immunoblots of three independent experiments are shown.

**Supplementary Figure 13.** **Expression of F-actin binding-deficient COTL1 mutant disorganizes the actin cytoskeleton assembly at the migrating wound edge.**

Fluorescence imaging of F-actin at the migrating edge of wounded DLD1 cells expressing control tGFP, tGFP-COTL1 WT, or tGFP-COTL1 R73E/K75E. Arrows point at prominent F-actin arcs in cells expressing wild type tGFP-COTL1. Arrowheads point at diffuse and diminished F-actin fibers in GFP-COTL1 R73E/K75E expressing cells. Representative of two independent experiments with multiple images taken per slide.

**Supplementary Figure 14. Overexpression of wild type COTL1 and its 5-LO uncoupled mutant similarly decreases IEC permeability and does not affect wound healing.**

(**A**) Immunoblotting analysis of DLD1 cells expressing either control tGFP, tGFP-tagged wild type COTL1 (tGFP-COTL1 WT) or COTL1 mutant unable to bind 5-LO (tGFP-COTL1 K131A). (**B**) Transepithelial electrical resistance and (**C**) transmonolayer FITC-dextran flux in DLD1 cells expressing control tGFP, tGFP-COTL1 WT or tGFP-COTL1 K131A mutant. (**D**) Wound closure of DLD1 cell monolayers stably expressing control tGFP, tGFP-COTL1 WT or tGFP-COTL1 K131A. Means ± SE (**B,C**: n =3; **D**: n=4); *p< 0.05 comparing to the control tGFP-expressing cells. Data are representative of at least two independent experiments.

**Supplementary Movie 1. Wild-type COTL1 and actin-binding deficient COTL1 mutants have different localization at the migrating cell edge.**

The movie shown fluorescence imaging of wounded DLD1 cell monolayers expressing either wild-type tGFP-COTL1 (a) or tGFP-COTL1 R73E/K75E (b).
